# Smart/stimuli-responsive hydrogels: Cutting-edge platforms for tissue engineering and other biomedical applications

**DOI:** 10.1016/j.mtbio.2021.100186

**Published:** 2021-12-09

**Authors:** Hussein M. El-Husseiny, Eman A. Mady, Lina Hamabe, Amira Abugomaa, Kazumi Shimada, Tomohiko Yoshida, Takashi Tanaka, Aimi Yokoi, Mohamed Elbadawy, Ryou Tanaka

**Affiliations:** a Laboratory of Veterinary Surgery, Department of Veterinary Medicine, Faculty of Agriculture, Tokyo University of Agriculture and Technology, 3-5-8 Saiwai Cho, Fuchu-shi, Tokyo, 1838509, Japan; b Department of Surgery, Anesthesiology, and Radiology, Faculty of Veterinary Medicine, Benha University, Moshtohor, Toukh, Elqaliobiya, 13736, Egypt; c Department of Animal Hygiene, Behavior and Management, Faculty of Veterinary Medicine, Benha University, Moshtohor, Toukh, Elqaliobiya, 13736, Egypt; d Faculty of Veterinary Medicine, Mansoura University, Mansoura, Dakahliya, 35516, Egypt; e Division of Research Animal Laboratory and Translational Medicine, Research and Development Center, Osaka Medical College, 2-7 Daigaku-machi, Takatsuki City, Osaka, 569-8686, Japan; f Department of Pharmacology, Faculty of Veterinary Medicine, Benha University, Moshtohor, Toukh, Elqaliobiya, 13736, Egypt

**Keywords:** Smart/stimuli-responsive hydrogels, Biomedical applications, Tissue engineering, Drug delivery, 3D printing, Biosensors

## Abstract

Recently, biomedicine and tissue regeneration have emerged as great advances that impacted the spectrum of healthcare. This left the door open for further improvement of their applications to revitalize the impaired tissues. Hence, restoring their functions. The implementation of therapeutic protocols that merge biomimetic scaffolds, bioactive molecules, and cells plays a pivotal role in this track. Smart/stimuli-responsive hydrogels are remarkable three-dimensional (3D) bioscaffolds intended for tissue engineering and other biomedical purposes. They can simulate the physicochemical, mechanical, and biological characters of the innate tissues. Also, they provide the aqueous conditions for cell growth, support 3D conformation, provide mechanical stability for the cells, and serve as potent delivery matrices for bioactive molecules. Many natural and artificial polymers were broadly utilized to design these intelligent platforms with novel advanced characteristics and tailored functionalities that fit such applications. In the present review, we highlighted the different types of smart/stimuli-responsive hydrogels with emphasis on their synthesis scheme. Besides, the mechanisms of their responsiveness to different stimuli were elaborated. Their potential for tissue engineering applications was discussed. Furthermore, their exploitation in other biomedical applications as targeted drug delivery, smart biosensors, actuators, 3D and 4D printing, and 3D cell culture were outlined. In addition, we threw light on smart self-healing hydrogels and their applications in biomedicine. Eventually, we presented their future perceptions in biomedical and tissue regeneration applications. Conclusively, current progress in the design of smart/stimuli-responsive hydrogels enhances their prospective to function as intelligent, and sophisticated systems in different biomedical applications.

## Highlights

• Different types of smart/stimuli-responsive hydrogels were outlined.
• Different approaches to synthesize and design smart/stimuli-responsive hydrogels were discussed.
• The exploitation of smart/stimuli-responsive hydrogels in diverse biomedical applications was highlighted.
• Current challenges and future directions for use of smart/stimuli-responsive hydrogels were presented.

## Graphical abstract

**Figure:**
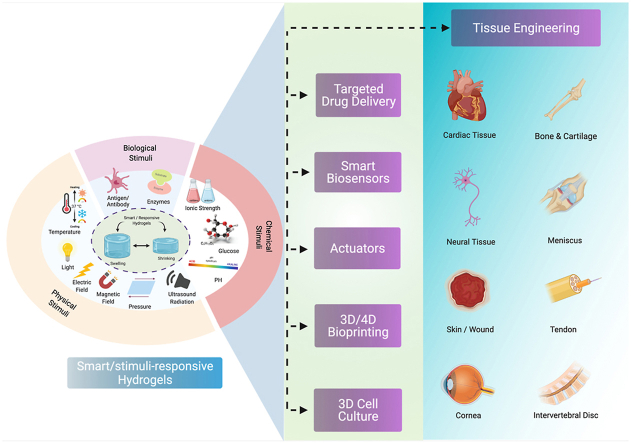

## List of abbreviations:

3D: Three-dimensional
Alg: Alginate
BASCs: Brown adipose-derived stem cells
CH: Chitosan
CMC: Carboxymethylcellulose
CMCH: Carboxymethyl chitosan
ECM: Extracellular matrix
GSH: Glutathione
FRP: Free radical polymerization
HA: Hyaluronic acid
Ins: Insulin
KGM: Konjac glucomannan
LCST: Lower critical solution temperature
Lir: liraglutide
MI: Myocardial Infarction
MMPs: Matrix metalloproteinases
NIR: Near infrared
NOCC: N, O-carboxymethyl chitosan
PAA: Poly(acrylic acid)
PAAm: Poly(acryl amide)
PBA-PGA: *γ*-Polyglutamic acid
PDGF-BB: Platelet-derived growth factor-BB
PEG: Polyethylene glycol
PEGDA: Polyethylene glycol diacrylate
PNIPAAm: Poly (N-isopropylacrylamide)
ppy: Polypyrrole
PVA: Polyvinyl alcohol
ROS: Reactive oxygen species
SPIONs: Superparamagnetic iron oxide nanoparticles
SWCNTs: Single-wall carbon nanotubes
US: Ultrasound
VEGF: Vascular endothelial growth factor

## 1 Introduction

The main objective of tissue engineering is the renovation of the injured tissues and replacing them with new biological ones [1]. This multidisciplinary process requires studying cell biology and biochemistry. Moreover, clinical medical and material sciences studies are incorporated for clinical applications [2].

The biologically active platforms are porous, three-dimensional (3D) structures that can support the attachment of biologically active components as biomolecules, proteins, and growth factors to their surface. The capacity of these biosystems to provide specific bioactivity to the assembly of the scaffold confirms their unique promises in tissue engineering and various biomedical applications [3]. They can function as cargos for the delivery of drugs and bioactive peptides, filling agents, and 3D structures. In addition, they can control the regeneration processes and promote the development of the required tissue [4]. Herein, the biomaterial must present the ideal characters matching the necessities of tissue regeneration. Cell seeded bioactive materials play a leading role in newly developed tissue formation through guiding self-seeded cell growth or stimulating cell migration. Besides, they act as cell delivery matrices to the targeted body tissues. Moreover, they mediate the fidelity of newly developed tissue structure and function [5]. For these purposes, they must have the fundamental physicochemical characters that provoke attachment of cells to their surfaces, cell growth, multiplication, differentiation, and migration [6], and to avert the unfavorable sequela, as cell necrosis and defective tissue regeneration, that usually encountered due to lack of these properties [3,6].

Throughout the years, several biological platforms have been fabricated from diverse natural sources like algae [6], and animal tissues [7,8], or synthetic sources like lactic acid [9], glycoside monomers [10], and caprolactone [11,12]. Even though many polymeric scaffolds used for tissue engineering could provide essential support and assets necessary for that purpose, they lack some crucial properties as adequate cell mimicking and sufficient interface with stromal cells. Scaffolds based on hydrogels could provide an adequate less invasive alternative that could support the properties missed in these scaffolds [3].

Hydrogels are hydrophilic 3D polymeric structures with their liquid fraction is water. They may be nature-derived, artificial, or semisynthetic. They are present in different body structures as extracellular matrix (ECM), epidermis, mucous, cartilage, meniscus, gelatin, collagen, tendons, and vitreous humor [11,12]. They were suggested to be novel materials with promising outcomes in the engineering of tissues. Owing to their hydrophilic structure, they can carry a massive quantity of water or biological fluids, within which the nutrients dissolve and diffuse to cells. Their cross-linking structure supports their integrity and prevents their disintegration within largely aqueous environments. Besides, they are supportive to the adjacent cells since they adopt a substantial level of elasticity and flexibility resembling the native ECM [13].

The ideal bioscaffold can bridge the tissue defects and enhance their reconstruction via provoking new tissue growth, neovascularization, and simultaneously showing high degrees of incorporation and biodegradation as they should disappear during or just after healing completion [14]. Owing to their distinctive structure and characters, hydrogels are considered the leading scaffold for different biomedical and tissue engineering applications [15]. However, rendering their clinical usage more significant is still challenging to make them more able to fit the continuous body functional and pathological alterations. Thus, the generation of novel hydrogel materials with smart properties able to promote clinical applications in biomedicine and tissue engineering is still required. Herein, researchers are increasingly recognizing that recapitulating the innate reactive ability of original tissues to biophysical and biochemical signals is a main feature for further upgrading functional tissue repair. This fast-growing concept is guiding the production of smart hydrogel platforms programmed with on-demand or stimuli-responsiveness [16,17]. Smart/stimuli-responsive hydrogels can present stimuli-induced volume and structural transitions, providing several multidimensional applications [18]. Generally, stimuli-responsive hydrogels can exhibit responses to fine environmental alterations like temperature [19], pH [20], ionic strength [21], other chemical stimuli [22], electric field [23], and biological circumstances [[24], [25], [26]]. According to their anticipated application, hydrogel platforms can be fabricated based on chemical and/or physical interactions. Chemically developed hydrogels depend mainly on a covalent crosslinking of their polymeric structure [27]. On contrary, physical interactions include the interaction between differently charged polyelectrolytes or between them and different charged polyvalent surfactants/ions [28,29]. These smart platforms can exhibit reversible swelling-deswelling transitions responsive to different stimuli [30]. These smart hydrogels can be synthesized by one, two, or various polymers to produce homopolymeric, copolymeric, or multipolymeric platforms, respectively [11,28]. Accordingly, they can afford diverse functionalities. Moreover, they can be designed in many preferred dimensions. For instance, they can fit many biomedical applications via amending their chemical make-up, configuration, biodegradability, biological moieties, and different physicochemical characters as rheological and mechanical, pH steadiness, spectral, loading, and discharge assets [31,32]. Such smart designs permit extraordinary surveillance over network assembly/disassembly, selective biomolecules presentation, and other adjustable properties upon realizing and responding to either intrinsic physiological or extrinsic applied stimuli [1]. This review will point to the evolution of hydrogel polymeric scaffolds in different biomedical applications with a special emphasis on tissue engineering. In addition to contemporary progress in smart/stimuli-responsive hydrogels and their future perceptions in regenerative medicine and other biomedical applications.

## 2 Conventional hydrogel polymeric scaffolds

### 2.1 Classifications of conventional hydrogels

Hydrogels could be classified based on different criteria including their polymeric composition (homopolymeric [33], copolymeric [34], multipolymeric [35,36]), and their configuration/physical structure (amorphous, semicrystalline, and/or crystalline). According to the type of crosslinking, hydrogels may be chemically crosslinked or physically crosslinked [35]. Chemically crosslinked hydrogels comprise the following subclasses; radically polymerized hydrogels, hydrogels based on the chemical reaction of the functional groups, high energy irradiated hydrogels, enzymatically crosslinked hydrogels, and special structural configurated hydrogels. The latter subclass involves side ring gels, double network hydrogels, and nanocomposite hydrogels). While the subclasses of physically crosslinked hydrogels include hydrogels based on hydrogen bonding interactions, freeze-thawing, thermogelation, charge interactions, and microgels and nanogels [37,38]. According to the physical appearance, hydrogels may be in the form of matrix, film, and/or microsphere. Based on the electrical charge of their network, hydrogels may be ionic, nonionic, amphoteric electrolyte, and/or zwitterionic charged [11].

Hydrogels are typically blended with various nature-derived, synthetic polymers, or a mixture of both. Alginate (Alg.), chitosan (CH), hyaluronic acid (HA), collagen, and gelatin represent the frequently used natural polymers. Synthetic polymers comprise poly(acrylic acid) (PAA), poly(acrylamide) (PAAm), poly(2-hydroxyethyl methacrylate), and polyethylene glycol (PEG) [39].

### 2.2 Challenges to use conventional hydrogels in biomedicine and tissue engineering applications

For decades, hydrogels were engaged in a broad spectrum of applications and have provided fascinating scaffold systems for the applications of tissue engineering [40]. However, the usage of conventional hydrogels is, in some instances, still encountered as an eminent challenge. For instance, many pristine hydrogels lack enough mechanical intensity [41], while stable, mechanically strong hydrogels are usually preferred for long-term, tension-bearing applications [42]. They exhibit spatial inhomogeneity, where the allocation of their crosslinking density is not homogenous, so lowers the potency of the hydrogel framework. Furthermore, the fragility of hydrogels makes their loading and manipulation difficult. As the hydrogels lack the adherence property, they may require to be supported by an additional dressing material. Also, their curative use for exudative wounds is not effective because of their limited absorption capacity [43].

The issue of hydrogel biodegradability and biocompatibility has attracted attention over the recent decades. Among the major advantages of degradable hydrogels decided for biomedical purposes was that they undergo hydrolytic and enzymatic degradation inside the body after achieving their target. Hence, no need to remove them from inside the body [4,40]. Regards synthetic hydrogel polymers, their restricted biodegradability, and biocompatibility rendered their usage in the reconstruction of different tissue somewhat challenging. During the evolution of hydrogel polymers, the degradation speed of hydrogels is owed to the proposed application. The biodegradable hydrogels that serve as temporary scaffolds are appropriate for cell culture, while those with low degradation profile are appropriate for applications of tailored biomolecules delivery where a longer time is compulsory to carry the drugs, nutrients, growth factors, peptides, and others to the targeted tissues [44,45]. Another constraint of conventional hydrogels is being difficult to be sterilized as they display a degree of sensitiveness to the traditional sterilization methods owed to their hydrophilic traits. The technicality of hydrogels crosslinking influences their release ability, where, the chemical cross-linking agents add another threat of toxicity [46].

The above-mentioned limitations have increased the research to adjust the hydrogels' properties and improve their position as an active system for different applications in biomedicine and tissue engineering. A novel property as electrical conductivity has been lately acquainted with hydrogels to broaden their pertinence and additionally the acknowledgment of new capacities while keeping up the initial ones (e.g., hydrophilicity and tenderness) [4,6,[47], [48], [49]].

## 3 Smart/stimuli-responsive hydrogels

**Fig. 1:**
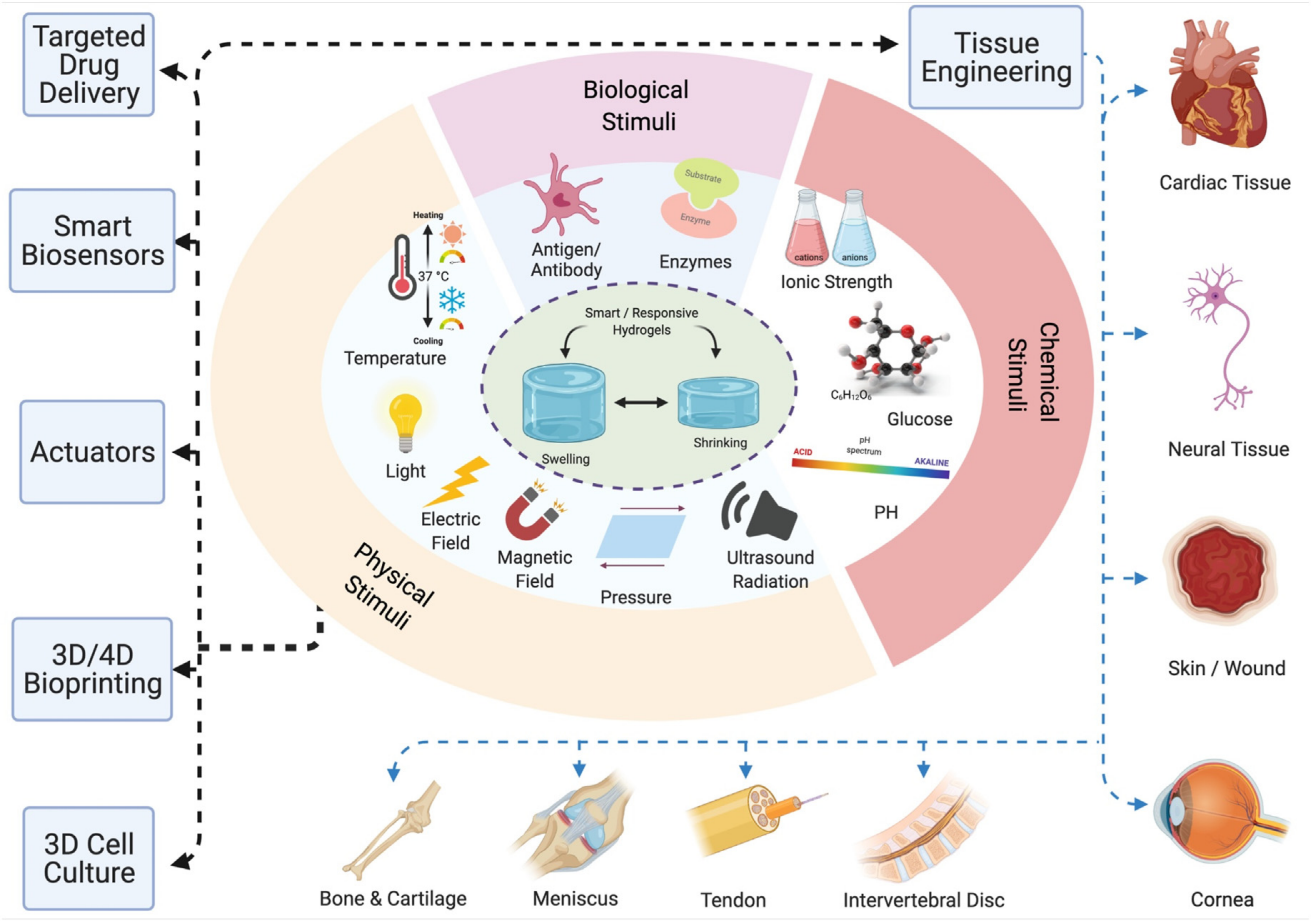
Schematic illustration of different smart/stimuli-responsive hydrogels employed for different biomedical applications.

Scientists have struggled to design hydrogels known as smart/intelligent hydrogels through amending their physicochemical features. These smart hydrogels can respond to various physical (temperature, light, electromagnetic fields, pressure, and ultrasound (US) radiation), chemical (pH, glucose, and ionic strength), or biological (enzymes and antigens/antibodies) stimuli and are described as stimuli-responsive hydrogels as illustrated in Fig. 1 [1,50].

### 3.1 General techniques for synthesis of hydrogels and smart/stimuli-responsive hydrogels

#### 3.1.1 Polymerization techniques

As 3D platforms, the hydrogel construction contains crosslinks that support their elasticity and viscoelasticity. Two sorts of monomers are included in the synthesis scheme of smart/stimuli-responsive hydrogels. The hydrophilic ones are exploited to fabricate the hydrogel, while those that are hydrophobic are important to adjust the hydrogel physicochemical and mechanical characteristics for definite applications. Synthetic polymeric hydrogels are characterized by being hydrophobic. Hence, they present a steadier chemical structure than natural polymers. Moreover, they present lower biodegradability, and robust mechanical assembly when combined with the natural ones [28,51,52].

The mixture of monomers that practice diverse responsive behaviors to various stimulations is the best strategy to design smart/stimuli-responsive hydrogels. To synthesize hydrogels, any approach that is appropriate for the production of cross-linked polymers is applicable. Free radical polymerization (FRP) methods are frequently employed with the polyfunctional cross-linkers to produce hydrogels from hydrophilic natural and/or artificial monomers [38]. The method of polymerization significantly affects the characters of the fabricated hydrogel [53]. The development of hydrogels could be accomplished either in an uni-step procedure via polymerization of the polyfunctional monomers with concurrent crosslinking of them or in a multi-step procedure that includes the assembly of reactive polymers that can be crosslinked by themselves or via reaction with suitable crosslinkers [38,54,55].

To synthesize composite hydrogels for certain applications, different inorganic, organic, and/or mixed polymers are combined to exploit the best assets of such materials. For instance, the parts of hydrophobic polymers were incorporated with proper crosslinkers to attain defined conformation and anticipated mechanical power [55,56]. The following is the discussion of different polymerization techniques employed for the fabrication of hydrogels. Table 1 presents the merits and demerits of each technique.

**Table 1:** Advantages and disadvantages of different polymerization techniques.

Polymerization Technique		Advantages	Disadvantages	Refs.
**Chain Growth Polymerization**	**Bulk Polymerization**	1. A simple method that involves only monomer and monomer-soluble initiators. 2. A high concentration of monomers enables a high rate and degree of polymerization. 3. Broadly applied to fabricate hydrogels. 4. Ability to control temperature and the concentration of the initiator. 5. Ability to control the conversion rate of bulk polymerization.	1. Generation of high temperature due to a marked elevation of the viscosity. 2. It is uneconomical in large-scale processes. 3. Poor mechanical properties of the hydrogels produced by bulk polymerization.	[38,57,58,59]
	**Solution Polymerization**	1. Facile synthesis. 2. Cost-effective. 3. High polymerization rate. 4. Can be conducted at room temperature. 5. Better control of heat transfer during the polymerization process than bulk polymerization. 6. Widely exploited for the synthesis of cellulose-based superabsorbent hydrogels. 7. The existence of solvent serving as a heat sink is the chief advantage of solution polymerization over bulk polymerization. 8. A preferred method for polymerization at laboratory and industrial scales.	1. Very sensitive to handle 2. Lack of sufficient reaction control over the reaction. 3. The reaction product sometimes is rubbery or solid. 4. Mono/poly-dispersity and an increase in the sol content mainly due to uncontrolled thermal and hydrolytic cleavage.	[60,57,61]
	**Suspension Polymerization**	1. Excellent heat transfer as water is the usual medium. 2. Inverse suspension polymerization is widely employed for the synthesis of hydrogels. 3. Grinding is not necessary for the dispersion polymerization method since the products are obtained as powder or microspheres (beads).	1. The dispersion is thermodynamically unstable 2. Continuous agitation and addition of a low hydrophilic–lipophilic-balance (HLB) suspending agent are requested.	[61,62]
	**Emulsion Polymerization**	1. Easily controlled. 2. Effective heat transfer over bulk polymerization.	-NA	[61]
	**Graft Polymerization**	-Improvement of the mechanical properties of the produced hydrogel more than bulk polymerization via grafting on a surface coated onto stronger support.	-NA	[38]
**Step Growth Polymerization**		1. Network cooperativity and homogeneity. 2. Ameliorated mechanical integrity, ductility, tensile robustness, and shear strain of step-growth polymerized hydrogels over chain growth polymerized hydrogels.	-Less rate of erosion due to exposure to light in chain-grown hydrogels because of the higher network connectivity.	[63]

NA, Not Applied.

##### 3.1.1.1 Chain growth polymerization technique

This type of polymerization with a free radical method is frequently exploited to fabricate chemically crosslinked hydrogels. The –C=C– involving hydrophilic monomers generally share in the FRP [60]. There are several approaches of FRP to develop hydrogels including bulk, solution, suspension, emulsion, and graft polymerization.

###### 3.1.1.1.1 Bulk polymerization technique

This method is broadly utilized to develop hydrogels because of its simplicity. That approach depends on the polymerization of fluid monomers and initiators soluble in monomers with little crosslinkers. The chemical catalysts, UV, and/or radiation are utilized to initiate the process of polymerization [57]. Shin and co-workers have developed bulk polymerized pH-sensitive hydrogels using acrylic acid-functionalized by sodium (NaAAc) and hydroxyethyl methacrylate (HEMA). Firstly, NaOH was utilized to neutralize AAc and obtain NaAAc. Afterward, the reaction was completed by adding α,α′-azobisisobutyronitrile (AIBN, the initiator), N, N′-methylenebisacrylamide (MBAAm, the crosslinker), and HEMA. The polymerization reaction was accomplished at 75 ​°C for 30 ​min and the components that were not included in the reaction were removed through repeated wash using deionized water [64]. To adjust the hydrogel characters, it is crucial to adjust the level of polymerization which is higher in bulk polymerization with lower efficacy to regulate the heat and quickly raise reaction viscosity. The produced hydrogel matrices are transparent and exhibit a high ability to swell and show high flexibility when immersed into water [38].

The emulsion polymerization method could yield smaller polymers than those constructed by the suspension method of polymerization. Furthermore, the emulsion and suspension polymerization procedures are more beneficial than bulk polymerization as they are simply tailored with better control of the produced heat [61]. The other polymerization approaches as solution, suspension, and emulsion techniques are superior to bulk polymerization. Hence, they are extensively exploited during hydrogel synthesis.

###### 3.1.1.1.2 Solution polymerization technique

To manufacture hydrogels using solution polymerization technique, the monomers (neutral or ionic), the solvent (for example, water, benzol, ethyl alcohol, or water-ethanol mixture), and polyfunctional cross-linkers are required to commence the polymerization by redox reaction or UV radiation. Eventually, the hydrogel polymer is parted, and the remaining components (cross-linkers, monomers, initiators, and other contaminants) are removed by distilled water wash [65]. The benefits of this technique include facile synthesis, improved control of heat transferal, safe, and cost-effective procedures [60,57].

It is frequently utilized to fabricate excellent absorbent cellulose-based hydrogel polymers. The polymerization method can be accomplished at ambient temperature with a high frequency of polymerization. Compared to bulk polymerization, mixing the solution polymerization reaction constituents is easier owing to the little solution viscosity. Hence, the dispersion of the heat dispersion in this technique is enhanced [60,61].

###### 3.1.1.1.3 Suspension polymerization technique

In this technique, the insoluble monomers, and the low hydrophilic-lipophilic equilibrium initiators are continuously stirred in a solution to produce 0.1–5 ​mm beads. With the advancement of the polymerization process, the hydrogel polymeric droplets are developed, then they are separated from the reaction components by filtration [66]. The heat transfer rate in this technique is elevated since water is the frequently used media. Moreover, colloidal agents like PVA, CMC, or methyl cellulose (MC) are utilized to prevent the adhesion between the droplets [61]. Furthermore, the reverse suspension polymerization technique is extensively exploited to produce hydrogels [62].

###### 3.1.1.1.4 Emulsion polymerization technique

This method is also utilized to fabricate hydrogels. The process encompasses water-soluble small monomers (totally hydrophobic or partially water-soluble monomers), a water-dissoluble initiator, crosslinkers, and a surfactant [65,67]. A hydrophilic organic monomer is applied in reverse emulsion polymerization [68]. The size of the polymeric droplets produced from this technique is less than that of polymers produced via the suspension technique. Like the suspension polymerization method, the emulsion technique is also easy to be adjusted and presents outstanding heat transfer capacity superior to that of bulk polymerization [61].

###### 3.1.1.1.5 Graft polymerization technique

Hydrogels synthesized via bulk polymerization present low mechanical abilities that can be provoked when utilizing graft polymerization technique, chiefly if grafted on robust support frameworks. The locations of free radicals are formed on the area of the support where the polymerization is achieved between the monomers to generate more potent covalent links with the support shell. For instance, vinyl monomers are usually grafted over polysaccharides [38]. Moreover, pH-responsive hydrogels produced via grafting of PAAc from CH hybrid with cellulose through formaldehyde resin are mechanically stronger than AAc grafted from CH hydrogels [69].

##### 3.1.1.2 Step-growth polymerization technique

To assemble hydrogels, certain monomers with definite functional moieties that can create covalent links to start an uni-step polymerization reaction are utilized in this technique [60]. The mechanical characters (integrity, ductileness, tensile strength, and shear strain) of light-degradable hydrogels developed via step-growth polymerization process were superior to those of hydrogels synthesized by chain-growth polymerization technique due to the cooperative and homogeneous traits of the network. However, the degree of degradation by light was lower for hydrogels.

Synthesized by chain-growth polymerization because of the strong linkage of their network [63]. The accurate perception of hydrogels crosslinking, and networking will help to synthesize hydrogels with anticipated characters.

#### 3.1.2 Crosslinking techniques

Crosslinking is imperative to prevent the disintegration of the hydrophilic elements of the hydrogel polymers in the aqueous media. Moreover, the more the cross-links the higher the hydrogel polymer elasticity and viscoelasticity [54]. The characters of synthesized hydrogels are highly correlated to the proportion of crosslinking [70,71]. Hence, the adjustment of crosslinking degree helps the production of hydrogels with diverse properties fit a vast spectrum of biomedical uses. Hydrogels are either physically (polymeric networks are synthesized via physical reactions including, for example, polycations-polyanions multivalent ionic reactions, or polymeric chain hydrophobic reactions) or chemically (polymeric networks are synthesized via chemical reaction as covalent bonds). Heat and UV radiation can trigger physical crosslinking, while several reactions including but not restricted to nucleophilic, Michael's, and Michaelis–Arbuzov reaction can achieve the chemical crosslinking [72]. Different crosslinking techniques are discussed below. Their advantages and disadvantages are presented in Table 2.

**Table 2:** Advantages and disadvantages of different crosslinking techniques.

Crosslinking approach	Advantages	Disadvantages	Refs.
**Physical Crosslinking**	1. No toxic crosslinking reagents were used. 2. An easy technique for hydrogel preparation.	1. Generally unstable and mechanically weak. 2. A limited number of compositions are suitable for physical crosslinking	[73,74]
**Chemical Crosslinking**	1. Resulted hydrogels are more stable and with better mechanical strength. 2. A high level of substrate specificity prevents unwanted reactions and the mild gelation conditions favorable for tissue regeneration (enzymatic crosslinking). 3. The latest advances in click chemistry offer highly orthogonal and selective interactions that efficiently progress under light circumstances. Moreover, these developments facilitate performing cellular functional tests on microenvironments.	1. The incorporation of chemical crosslinkers may cause toxicity problems (using toxic copper for click chemistry technique). 2. Full control over the final product is difficult owing to various termination 3. Mechanisms (Radical polymerization). 4. Biological compounds can pose the risk of side reactions by competing with nucleophiles (Michael-type reactions). 5. Low cellular efficiency, long reaction time, and extreme reaction conditions of the conventional chemical crosslinking techniques	[[75], [76], [77], [78], [79]]
**Hybrid Crosslinking**	-Merge the merits of both physical and chemical crosslinking and avoid their limitations.	NA.	[80,81]

NA, Not Applied.

##### 3.1.2.1 Physical crosslinking

Physically crosslinked hydrogels are synthesized through various reactions as ionic/electrostatic reactions, hydrophobic reactions for amphiphilic polymers self-assembly, hydrogen bonding, protein interactions, metal coordination, and crystallization. This interplay among the polymer chains does not include covalent links formation and synthesize phase-reversible hydrogels [82]. For instance, via hydrogen bonding, polymethacrylate and polyacrylate form compounds with PEG in presence of carboxylic acid groups protonation with consequent formation of pH-responsive hydrogel [83]. Likely, via ionic reactions with Ca^2+^ or Mg^2+^, the natural Alg. polymers create gels [82].

Furthermore, poloxamers as amphiphilic polymers form thermal-responsive hydrogels because of the hydrophobic reactions above LCST [84]. An example of crystallization prompted physical crosslinking are PVA-based hydrogels where the crystallization and gelling of the polymers are achieved with repeated cycles of freezing and thawing [85]. The major limitation of physically crosslinked hydrogels is their diminished or zero stability under physiological circumstances. Hence, chemical crosslinking is the alternative solution favored for applications in vivo [83].

##### 3.1.2.2 Chemical crosslinking

Unlike physical crosslinking, hydrogels produced by chemical crosslinking present higher stability under physiological states with outstanding mechanical characters because of the interpolymeric chains' covalent links. Moreover, they exhibit adjustable biodegradation although being irreversible. On the other side, many environmental and biocompatibility concerns have been raised owing to the increased need to utilize organic catalysts and solvents in chemical crosslinking [82]. Click chemistry has promoted the covalent links in the hydrogels developed by chemical crosslinking through several reactions as Diels–Alder reaction or Michael addition reaction, FRP, Schiff base formation, photopolymerization, enzyme-catalyzed reactions, or reversible addition-fragmentation chain transfer (RAFT) polymerization. The existence of definite groups like COOH-, OH-, and NH_2-_, in the assembly of the hydrophilic polymers, to construct the hydrogel, the amine-carboxylate, Schiff base creation or isocyanate-OH/NH_2_ reaction are generally exploited to form covalent bondage between the polymers [82]. Schiff base is the most widely utilized to develop hydrogels which are attained through the attack of amines on the electrophilic ketones or aldehydes carbon atoms. For instance, glycol CH was crosslinked with PNIPAAM-co-PAA or PEG to synthesize unique hydrogels that function as cargoes for the release of anticancer therapeutics to the place of tumors [86].

Glycidyl methacrylate (GMA) and Poly (ethylene glycol) diacrylate (PEGDA) as acrylic acid derivatives comprise unsaturated groups were utilized for hydrogel synthesis by photopolymerization crosslinking. Upon disclosure to visible light and/or UV, the light-initiators (lithium phenyl-2,4,6- trimethyl-benzoyl phosphinate (LAP), Irgacure 2959, Eosin-Y, or others) react with these unsaturated groups. Consequently, light-initiators are split by photons producing free radicals which interact with the pre-polymer vinyl bonds enhancing the polymeric chains crosslinking [87].

Lately, diverse enzymes have been exploited to fabricate hydrogels [88,89]. Enzymatic crosslinking provides a satisfactory degree of substrate specificity, hence avoiding undesired reactions. Besides, the light state of gelation is convenient for tissue engineering [73]. Horseradish peroxidase (HRP) was exploited to catalyze the hydrogen peroxide-based oxidation of several types of polymers with aminophenol, tyramine, or tyrosine side groups to synthesize in-situ forming hydrogels [[90], [91], [92], [93]]. To synthesize PEG-based polymeric hydrogels, transglutaminase enzyme was utilized to produce an amide link via catalysis of the poly(lysine-co-phenylalanine) ε-amine group reaction with the γ-carboxamide group of the glutaminyl groups functionalized PEG [28].

##### 3.1.2.3 Hybrid crosslinking

Hybrid crosslinking has been utilized to synthesize hydrogels that merge the advantages of physical and chemical crosslinking and avoid their disadvantages [80,81]. So that hybrid crosslinking developed hydrogels have currently attracted great attention for uses in biomedicine and tissue engineering. For instance, Shachaf and co-workers, via FRP utilizing photo-induced chemical crosslinking to conjugate fibrinogen to pluronic F127, developed a temperature-responsive hydrogel [94]. Moreover, a novel thermo-responsive CH-based injectable hydrogel was assembled by combined covalent and ionic crosslinking. Results revealed that gel transition following solution hydrogel injection has been achieved promptly and sustained for one week as a minimum [95].

Tissue engineering intended temperature-responsive hydrogels with intense mechanical properties were produced via the combination of native chemical ligation (NCL)with thermo-stimulated crosslinking. To produce this hydrogel polymer, N-(2-hydroxypropyl)-methacrylamide-cysteine (HPMA-Cys) monomer was polymerized with NIPAAm. Furthermore, thioester cross-linkers were fabricated by PEG and HA. The findings also showed that PNIPAAm chains self-assembly property has stimulated gel transition with the raise of temperature to 37 ​°C. For the produced hydrogel polymer to be stronger and steadier, covalent crosslinking of cysteine and thioester was achieved [95].

Dynamic covalent bonding is being adopted to attain hybrid hydrogels that merge the merits of both chemical and physical crosslinking with higher mechanical assets and enhanced reversibility. For instance, boronic acid esters obtained from 1,2- or 1,3-diols and boronates in hydrous solutions produce reversible covalent links. Since the development of boronic acid ester is preferred at pH over the pKa of the boronate, the reversibility and strength of the produced bonds are correlated to the solution pH and the pKa of the boronate. Hence, amendment of the used 1,2- or 1,3-diols and boronate derivative results in the production of pH-responsive hydrogels with unique reversibility and adjustable mechanical characters [82].

### 3.2 Types and synthesis of different smart/stimuli-responsive hydrogels

#### 3.2.1 Physical stimuli-responsive hydrogels

##### 3.2.1.1 Temperature/thermo-responsive hydrogels

Amongst other categories of smart/stimuli-responsive hydrogels, thermo-responsive hydrogels are the most investigated. Hydrogel solutions are featured by possessing a lower critical solution temperature (LCST). They are inverse temperature-dependent; they shrink when the temperature exceeds the LCST [96]. Many polymers with LCST are currently employed for different biomedical applications. Among them are PEG (106–115 ​°C), poly(propylene glycol) (PPG) (10–40 ​°C), PVA (125 ​°C), PNIPAAM (32 ​°C), poly(methyl vinyl ether) (PMVE) (28–34 ​°C), and poly(N-vinyl caprolactam) (PNVCa) (30–50 ​°C) [97].

**Fig. 2:**
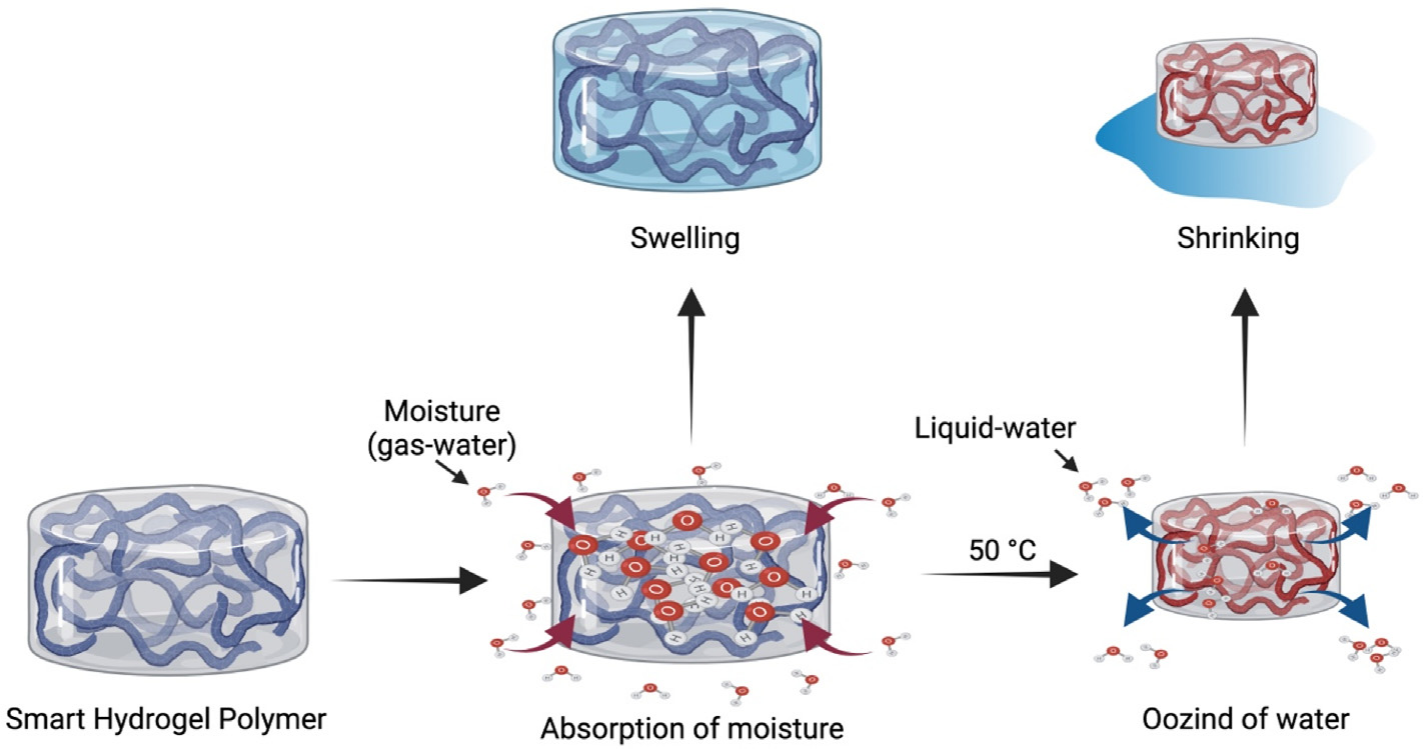
Thermo-sensitive swelling-deswelling behavior in temperature-responsive hydrogels.

**Fig. 3:**
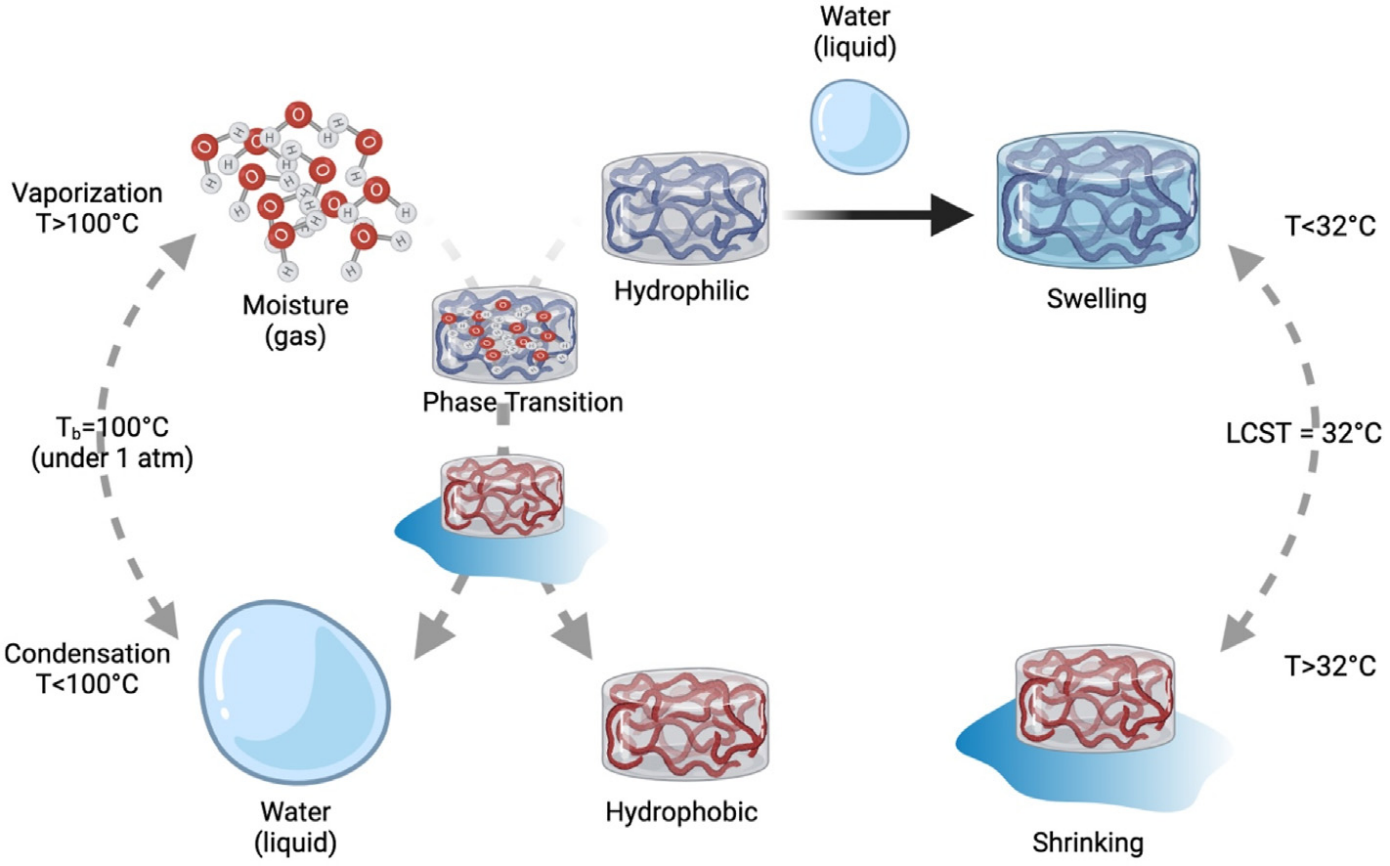
Schematic illustration of water absorption and leaching behavior of dried PNIPAAM hydrogels.

The capability to combine several polysaccharides (e. g., CH, chondroitin sulfate, HA, PEG, Alg., dextran, and cellulose) with thermo-responsive hydrogel polymers will pave the way to introduce novel hydrogels with promising properties applicable for tissue engineering [[98], [99], [100]]. Temperature-responsive hydrogels are distinguished by containing hydrophobic moieties as propyl, ethyl, and methyl groups [101]. Multiple natural (cellulose, CH, and gelatin) and synthetic (PNIPAAm, and polyfluorene 127) polymers are exploited to synthesize thermo-sensitive hydrogels [102]. Since the hydrogel responsive behavior is controlled by the kind of the monomer and the crosslinker, the monomer should be precisely chosen during the hydrogel synthesis. NIPAAm is the widely exploited monomer to fabricate hydrogels for drug release intentions. PNIPAAM is amongst the most frequently investigated hydrogels, particularly for applications in biomedicine as their volume phase transition temperature (VPTT) at LCST is closer to the human temperature and they possess quick on-off switching abilities [103,104]. PNIPAAM polymers experience a coil-to-globule transition via contraction of their polymeric chain because of the hydrophobic influence and a raise in entropy of the system [105]. Although the polymer exhibits a sol-to-gel transition, the adjacent water molecules can freely move with the coil-to-globule transition, hence elevating the net entropy of the system. Studies declared that the existence of amide hydrophilic groups and isopropyl hydrophobic groups in the side chains enables PNIPAAm to exhibit temperature-responsive phase alteration behavior in hydrous solution at LCST of about 32 ​°C [98,106,107]. Many in-depth studies have been conducted to elaborate on the mechanism of LCST polymers. Also, they provided further information about the thermogelling polymers and inspire the fabrication of temperature-responsive hydrogels. For example, the copolymerization of PNIPAAM with hydrophobic/hydrophilic monomers can adjust the LCST value of PNIPAAM. The Addition of hydroxylethylacrylamide (HEAm) hydrophilic monomers could increase the LCST to 50 ​°C [108] as illustrated in Fig. 2. In contrast, the addition of N-tert-butylacrylamide (NT), tertbutyl acrylamide (TBAM), or butylacrylate (BA) hydrophobic monomers to PNIPAAM could lower its LCST and produce a stiffer hydrogel [109,110]. Moreover, the affinity of dried hydrogels to water drastically changes to minor alterations in the temperature. The hydrogels absorb the molecules of water in their gaseous form (at 100 ​°C, and 1 atmospheric “atm” pressure). However, it was oozed out from the dried hydrogel in its liquid form. This mechanism was attributed to the alteration in the hydrophilicity/hydrophobicity of the hydrogel chains as illustrated in Fig. 3. BC and castor oil combined with NIPAAm represent renewable resources to synthesize novel temperature-responsive hydrogels [111].

Some bioactive materials based on cellulose have been utilized for the engineering of nerve tissues [112]. Likely, CH/collagen, CH/CMC, and CH/PEG have been exploited to synthesize temperature-sensitive hydrogels for use in invasive surgeries [[113], [114], [115]]. Besides, they can serve as potent injectable drug vehicles that present temperature-responsive gelling properties [116].

Temperature-responsive hydrogels synthesized from MC, collagen, agarose, and block polymers of PEG/polyethylene oxide (PEO) in addition to gelatin, pluronic, poly(N-PNIPAAm) and their derivatives fit the sophisticated applications of bioprinting [117]. They exhibit outstanding temperature-dependent sol-gel transition capacity, unique resolution, precise printability [118], cell biocompatibility [119,120] with the capacity to imitate the tissue micro-environment, vascularity, and native shape [121,122].

Thermo-responsive hydrogels were designed as cargo for tailored drug delivery at normal body temperatures (36–37 ​°C). Upon exposition to localized external heating, these drugs are rapidly released [96]. Researchers have fabricated thermo-responsive lysolipid liposomes that open their membranes under exposure to mild hyperthermia (i.e., 41–43 ​°C) [123,124]. They merged lysolipid liposomes loaded with doxorubicin within injectable CH/β-glycerophosphate hydrogel systems. Under 42 ​°C hyperthermic pulses, they displayed a 7-fold increased drug release [123]. O'Neil and coworkers utilized thermo-responsive hydrogels as proangiogenic therapeutic scaffolds. In this investigation, deferoxamine-loaded liposomes, and hepatocyte growth factors were entrapped in a CH-based hydrogel matrix to accomplish a consequent release of multiple biomolecules [124]. The profile of deferoxamine release was further controlled by suspending the hyperthermic stimuli. This process has promoted the recruitment of stem cells because of the erupted liberation of the chief growth factor plus the subsequent on-demand postponed release of pro-angiogenic deferoxamine under the impact of external hyperthermic stimuli.

To intensify the threshold response levels or allow on-demand discrepancy actuation of the discrete constituents. More future research endeavors should direct toward simultaneous integration of thermo-responsive hydrogels with overlapping temperature triggers or encoded with differential thermal behaviors respectively. Besides, thermo-responsive scaffolds are commonly incorporated as essential actuators in designing advanced hybrid external stimuli (photo, magnetic, and US)-responsive platforms.

##### 3.2.1.2 Light/photo-responsive hydrogels

Light-sensitive hydrogels are those hydrogels that respond upon exposure to light stimuli. They comprise a polymeric system and a functional photoreceptive moiety. Their physicochemical characteristics change upon light exposure [[125], [126], [127]]. They are appealing for multiple applications in biomedical sectors, fundamentally if obvious light is utilized. Once the photochromic components receive the optical stimulus, the conversion of the photo-radiation to a chemical stimulus is facilitated by a photoreaction including isomerization, cleavage, and/or dimerization. This signal is then directed to the hydrogel functional part and influences its properties [128]. Light-responsive hydrogels comprise two main classes: the first one encloses photo-sensitive moieties (e.g., o-nitro benzyl and azobenzene), while the second one encloses NIR absorbing nanostructures (e.g., nano-shells, nanorods, and carbon nanotubes incorporated in temperature-sensitive hydrogels) [129]. These polymeric hydrogels function as photo-responsive systems via embedding of photochromic particles in the hydrogel set-up that can be accomplished through mechanical methods or chemical combination. This capacity is tuned through a precise assortment of chromophores, light intensity, wavelength, and chromophore-polymer communications [130]. Upon disclosure to light stimuli, these hydrogels exhibit sol-to-gel transitions consequent to the splitting of light-sensitive moieties connected with the hydrogel platforms. Moreover, they present either chemical alterations [128] or photothermal induced swelling-deswelling behavior because of the heating of nanoparticles integrated within thermo-responsive hydrogel matrices [131].

Studies on photoreactive groups, cyclodextrin (CD) or azobenzene-modified dextran, containing photo-responsive hydrogels as protein conveyance platforms revealed that hydrogels sensitive to certain molecules have a remarkable tendency to be operated as tailored drug-carrying devices [132,133]. Another investigation proposed that PNIPAAm composites developed from glycidyl methacrylate (GMA) nanofillers and graphene oxide (GO) exhibited photo-sensitivity due to GO responsiveness to the infrared light [134]. Lately, another light-responsive nanocomposite hydrogel incorporated within a temperature-sensitive and mechanically strong dual networked matrix was synthesized [131]. Besides, photo-responsive hydrogels founded on light-sensitive components as polydopamine [135] and semi-conductive polymers [136] were designed.

Yet, the responsive period of these hydrogels is too long. Therefore, important enhancements in their properties are essential to obtain quick responding hydrogels [137]. The usage of photo-sensitive hydrogel scaffoldings in biomedical implementations is limited as most of them include groups reactive to UV. Nevertheless, they possess a special potential in the applications of precise drug transport as the photo stimulant can be instantly and perfectly imposed [96].

Recently, hybrid PEG-based hydrogels with light-activatable cell-attached motifs were developed to provide elegant 4D platforms capable to modulate embedded human vascular endothelium responses as cell adherence and angiogenesis [138]. These hybrid hydrogel systems were integrated with upconversion nanoparticles.

In other experiments, upconversion nanoparticles were inimitably important to support tissue-permeable optogenetic regulation through activation of light-sensitive proteins in engineered cells or provoking localized siRNA delivery to curb the biological functions [[139], [140], [141]]. This unique class has also effectively extended the light sensitiveness of the immunomodulatory agents activatable by UV light to deep tissue penetrating near-infrared (NIR) light in cancer in vivo immunotherapy approaches [142].

##### 3.2.1.3 Electric/magnetic (electro-magnetic)-responsive hydrogels

Electro-magnetic-responsive hydrogels are those hydrogel systems that are reactive to minor electrical and magnetic field alterations with consequent change of their characters (shrinking, swelling, or bending). These systems were typically investigated as ionizable rich polyelectrolyte hydrogels [96,143].

**Fig. 4:**
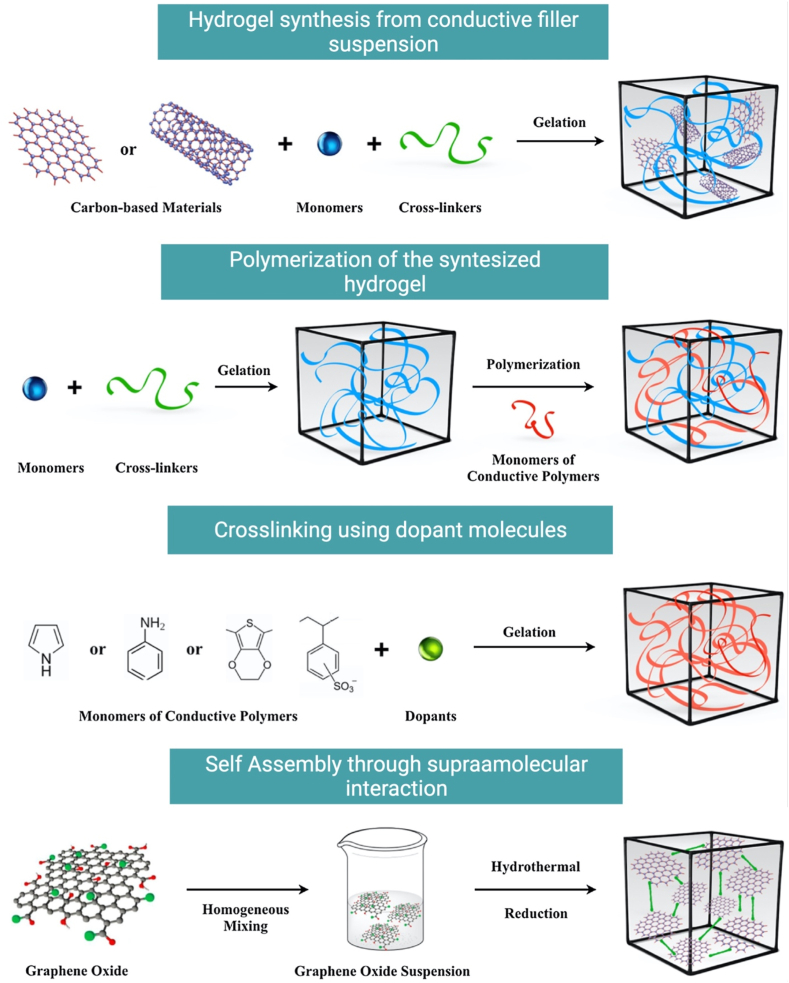
The most employed techniques to synthesize electro-responsive hydrogels. Copyright © 2021, ELSEVIER Publishing Group. Replicated with permission from Ref. [148].

Several kinds of polymers were used to develop electroactive and magnetic-sensitive hydrogels. These polymers were either synthetic including, for example, polyvinyl alcohol (PVA), sulfonated polystyrene, or acrylate/vinyl sulfonate, or natural such as Alg., HA, and CH. Natural polymers were mingled with synthetic ones to produce unique mixed systems of hydrogels [144,145]. FRP and chemical crosslinking were exploited to synthesize electro-responsive poly(2-(acrylamide)-2-methylpropanesulfonic acid) (PAMPS) hydrogels [146,147]. Electro-conductive materials as conductive polymers including polypyrrole (ppy), poly-3,4-ethylene dioxythiophene (PEDOT), polyaniline (PANI), and others, and metallic nanoparticles like Au nanoparticles, and carbon-based nanoparticles like graphene and carbon nanotubes are usually incorporated in the hydrogel matrices to synthesize electro-responsive hydrogels [23,148] as illustrated in Fig. 4.

Recently, electro-responsive hydrogel systems composed of photopolymerizable polyacrylamide/CH hydrogel networks entrapping ppy nanorods have been consumed as robust electroactive nanocomposite hydrogels that could provide electro-responsive dexamethasone delivery. Besides, enhancement of myoblast proliferation of C2C12 mouse even with lack of external electrical stimuli [149]. Besides, the authors tested their capacity to augment the reconstruction of full-skin defects in wound models of rats. The loading of epidermal growth factors to this hydrogel system has further boosted the healing operation of the epidermal tissues [150]. Otherwise, polyaniline-based hydrogel matrices have been broadly employed in a lot of biomedical applications [151]. For instance, polyaniline nanowires have been embedded in gelatin hydrogels to construct electroactive scaffolds that were used for bioprinting complex, and user-defined constructs containing osteoblast-like cells [152]. Currently, some researchers have reported that naïve stem cells could be stimulated to the osteogenic lineage using electrochemical signals. This presents important implications regarding stem cell superiority in standard cell-based therapies and bottom-up tissue engineering implementations [153,154]. Furthermore, electrical stimuli could influence cell processes in angiogenesis, neurogenesis, or cardio-myogenesis, and were utilized for tackling cell-derived electrochemical correspondence to inactively upgrade microtissue development over time [2]. These findings support some cell lineages' reliance on 3D electroactive scaffolds for spreading fundamental bioelectrical signs, therefore enhancing bio functionality in engineered tissues. Soon, bioelectronic interfaces that are exceptionally subject to it will be intriguing to follow the value of such stimuli-responsive hydrogels in the cutting-edge development of useful electro-responsive hybrid scaffolds [155].

Magnetic nanoparticles are usually incorporated within the crosslinked polymers to synthesize magnetic responsive hydrogels [156]. To synthesize magnetic-sensitive nanocomposite hydrogels, three major techniques are utilized including the co-precipitation or encapsulation approach, the grafting method, and the blending approach [[157], [158], [159], [160]]. The magnetic portion acts as a composite inside the hydrogel framework so that upon magnetic stimulation, the thermal, mechanical, and acoustic states are instantaneously disturbed [157]. The magneto-thermal capacity of magnetic-responsive hydrogel polymers can be managed through the adjustment of the magnetic field to control the organization of the magnetic nanoparticles within the hydrogels [158,[161], [162], [163]]. These nanoparticles can be constructed into an uni-dimensional configuration to enhance their homogeneity, mono-scattering, and their overall magnetic characters [164,165]. Magnetic-responsive hydrogels could be synthesized using suspension polymerization depending on one of these four main techniques: mixing and gelation, mineralization, coupling, or simultaneous formation [[166], [167], [168]].

Magnetic guidance of nanoparticles within nanocomposite hydrogels under stationary magnetic fields has been exploited as an outstanding drug delivery trigger where the mass directional motion causes mechanical deformity of hydrogel networks resulting in enhanced drug release [1]. For example, Pluronic F127 hydrogels were utilized as magnetic responsive indomethacin release systems. The distinctive sol-gel transition character rendered this ferrogel a successful injectable drug cargo that required no further surgical implant procedure. In this work, indomethacin release was enhanced in the existence of magnetic stimuli, reaching (50% release after 25 ​h), whereas this release rate was slower in the absence of the extrinsic magnetic fields (only 15% release after 25 ​h) [169]. Augurio et al., have incorporated SPIONs filamentous nanoparticles within methacrylate gelatin-based magnetic-responsive hydrogels to boost the magnetic guidance property and enable real-time remote guidance of constructs post-implantation [170].

Different studies have reported that, upon magnetic stimulation, magnetic nanoparticles inserted in collagen-based hydrogels/bioinks could successfully achieve unidirectional orientation of collagen fibers with an overall enhancement of their mechano-compressive strength, and guiding cell alignment with the marked promotion of many cell activities including, for example, enhanced ECM production, biochemical signal propagation, neuro- and myogenesis [171,172]. On the other hand, a two-photon polymerization approach was employed to attach magnetic beads (microactuators) to the PEDGA hydrogels. The linked microactuators presented designed 2D and 3D shape transitions with substantial actuation of the microstructured hydrogels. Besides, this fabrication technique enables the designing of soft micromachine with unique applications at the cell level [173]. In another work, a magnetic-responsive nanocomposite hydrogel system (NIPAAm–AAm–PEDGA and MNPs) was developed based on photolithography [174]. The developed micromachines presented exceptional magneto-sensitive shape transformations and adjustable motility with promising potential to broaden the scope of biomimicry to synthetic microbes. Goudu S. et al. [175], designed a biodegradable and successfully biocompatible magnetic-sensitive hydrogel system based on photopolymerized swine ECM collagen and SPIONs dispersed in hydrogel milli-gripper polymeric precursor liquid exposed to an extrinsic magnetic field. The facile fabrication technique to design such magneto-sensitive nanocomposites is highly promising for diverse future biomedical applications. In another sophisticated work, Tasoglou S. and co-workers [176] could utilize the paramagnetic fields to control the assembly of MNPs-free hydrogels via levitation. This unique approach assisted to avoid the hazard effects of MNPs on the cells specially for tissue engineering purposes, the magnetic inconstancy, and the clustering of MNPs.

Magnetic-responsive agarose hydrogels entrapping glycosylated SPIONs were also utilized to convey growth factors and other molecules to osteochondral tissues. In addition to establishing complex biochemical gradients in cell-loaded hydrogels under the impact of magnetic fields, as a path to develop osteochondral interfaces that imitate the parental tissue growth factor gradients [177]. Moreover, different magnetic-responsive hydrogels have been exploited for cell [178], and drug [179] delivery.

However, with the great promises they exhibit, these scaffolds usually lack adequate mechanical firmness. Hence, improvement of their mechanical characteristics was attractive to make benefits, especially when intended to be administered in load-bearing tissues and where the layout of mechanically robust hydrogels with double networks is highly pursued [180].

Recently, Tang et al. could successfully design double-network hydrogel systems combined with magnetic nanoparticles. These platforms exhibited excellent mechanical power and could be utilized to improve the control of hyperthermia and accomplish controlled drug release [181].

##### 3.2.1.4 Pressure/strain-responsive hydrogels

Pressure-responsive hydrogels have a novel capacity to exhibit responses to the recognized compressive stimuli which is more obvious in cellular designed, nanofibrous, and hyper-elastic hydrogels. For instance, hydrogels fabricated through a combination of Alg. and malleable SiO_2_ nanofibers. Owing to their very high amount of moisture (over 97%), hydrogels do not present strong mechanical capacity and meaningful reversible deformity. Such hydrogels are comprised of several polymeric components as nanoparticles, polyelectrolytes, proteins, polysaccharides, and others, into 3D frameworks. In water-rich conditions, they lack the mechanical potency however their networks are homogenously organized. In this situation, hydrogels of fibrous cellular frameworks can exhibit facile promotion of their mechanical capacity with substantial pressure-responsive ability [182,183]. Upon disclosure to extrinsic mechanical stimuli, pressure-responsive hydrogels exhibit responsive swelling-deswelling and/or extension-shrinkage. In an elegant study, the mechanical shearing approach was utilized to synthesize quasi ester nano crystallized cellulose (CNC) through UV-induced polymerization. This polymeric hydrogel exhibited responsive swelling and quick reversible color alterations triggered by the pressure stimuli [184,185]. In an additional investigation, SiO_2_ nanofibers, Alg., water, and a metallic cation (Al^3+^) were combined by sol-gel electrospinning method to synthesize SiO_2_ nanofibrous hydrogels [186].

Recently, pressure-responsive hydrogels have revealed a substantial advance for usage in biomedical applications [187,188]. They represent a special kind of hydrogels that present essential flexibility, great sensitivity, and excellent repeatability which ensure their implementation both in single and cyclic pressure measures. These unique hydrogel systems usually present responsive modifications in their characters to external stressors. Also, the extent of such stressors and the action of external objects can be revealed by evaluating systems’ variations [189]. Zhu et al. [190] developed a remarkable promising hybrid hydrogel that can be employed as a pressure sensing device that can be worn to record the body activities ranging from big pressure to fine strain change produced during breathing. This novel ionic conductor smart hydrogel was formulated with diffusing sodium carboxymethylcellulose (CMC) micro-sheets in a PAAm polymeric network. Their interaction confirms an elegant mechano-physical firmness to the entire system, besides elevated adhesion efficacy, great sensitivity, high repeatability, and excellent genuine response. To prevent evaporation of the moisture content, the center of the sensor, fixed and encapsulated in an elastic substrate. On other side, the hydrogel-attached copper foil ensures electrical connection.

Xia et al. developed a highly efficient wearable biosensor using a hydrogel with remarkable ion conductivity and depending on a micellar-copolymerization process. The final system presented outstanding mechanical characters resembling that of skin with low modulus, high strength, and robust elasticity. Thanks to hydrogel responsiveness to extrinsic stimuli, great durability, and reproducibility, this device is suitable as a pressure sensor. Yet, this system can be used to monitor the motions and activity of humans [191].

As an intelligent stress sensor, Zhang et al. fabricated a composite hydrogel containing MXene that was possible to provide more than 14 folds extension of the hydrogel and reach a tensile power of 0.4 ​MPa while the entire system was demonstrated to be compact and resistant [192]. MXenes are a category of 2D inorganic compounds that attract extensive attention with their stress-sensing properties. In that device, MXene nanosheets have been encapsulated inside a matrix developed with poly(N-isopropyl acrylamide) (PNIPAAM) and a physical cross-linking hydrogel.

Mechanical forces have been exploited as triggers for controlled bioactive load transfer in the form of wearable strain-responsive scaffolds. Especially, poly-l-lactic-co-glycolic acid (PLGA) nanoparticles were entrapped in Alg.-based hydrogels. These hydrogel composites, as wearable devices, were employed as vehicles to deliver many therapeutics as ciprofloxacin, doxorubicin, and insulin (Ins.) for control of bacterial infections, cancer, and diabetes, respectively [193]. HA-based hydrogel microneedles were employed as an Ins.-releasing system to control diabetes. After in vivo administration, Ins. Plasma concentration increased with subsequent reduction of blood glucose concentrations. These systems could provide either i) sustainable release responsive to mechanical movements of muscles, tendons, and joints or ii) controlled, targeted pulsatile release (i.e., based on hand motions or other physical activities), to deliver antibacterial agents, Ins., or painkillers to the skin of patients [1]. Korin N. and co-workers [194] exploited shear-responsive PLGA nanomaterials as drug delivery cargos. They served as platelet mimetics and tailored the discharge of definite thrombus busting molecules (tissue plasminogen activator (tPA)) to the seat of vascular clot in response to the regional mechanical stimuli.

Anticipating future perspectives, further investigations considering the design are still necessary to boost the effect and translational capability of this exceptional class of stimuli-responsive hydrogels. For example, since drug discharge rates are correlated to the degree of the applied pressure, mechano-responsive biomaterials should provide therapeutic modalities at feasible and non-destructive mechanical forces.

##### 3.2.1.5 Ultrasound-responsive hydrogels

US-responsive hydrogels display responses to sound stimuli or US stimuli that are present beyond the level of human recognition [195,196] and possess diverse applications in several fields as mixing, cleaning, and imaging [197,198]. Their implementations with hydrogels are common in the sector of biomedicine including drug transport and cancer therapy [196,[199], [200], [201]]. When aroused by the US stimuli, they show a reversible de-crosslinking-crosslinking property. This renders the self-healing capacity to US-responsive hydrogels as their crosslinks disrupt upon disclosure to US stimuli and contrarywise, renewed when the stimuli are withdrawn. This process is employed to attain precise drug release devices [202]. The method to synthesize US-responsive hydrogels is correlated to their intended applications. For instance, Zhang et al. synthesized an MXene (Ti_3_C_2_T_x_)-based nanocomposite hydrogel, through the combination of MXene (Ti_3_C_2_T_x_) nanosheets, water, PVA, and anti-desiccation compounds, with outstanding ability to monitor the sound stimuli of human voice [203]. In another study, Kwok et al. has developed US-responsive hydrogel, which functions as drug distribution cargoes, through the formation of methylene chain in the Ins. Polymer. The crosslinked hydrogel polymers were produced via polymerization of HEMA monomer, PEG, dimethacrylate (PEGDMA), 2-hydroxyethyl acrylate (HEA), and Ins [204]. To enable the hydrogel platform to carry huge molecules of protein as Ins., PEG was utilized to produce porous matrices. Chains of methylene were synthesized on PHEMA via combining it, dibutyltin dilaurate (catalyst), C-12 isocyanate, and anhydrous tetrahydrofuran [204,205].

**Fig. 5:**
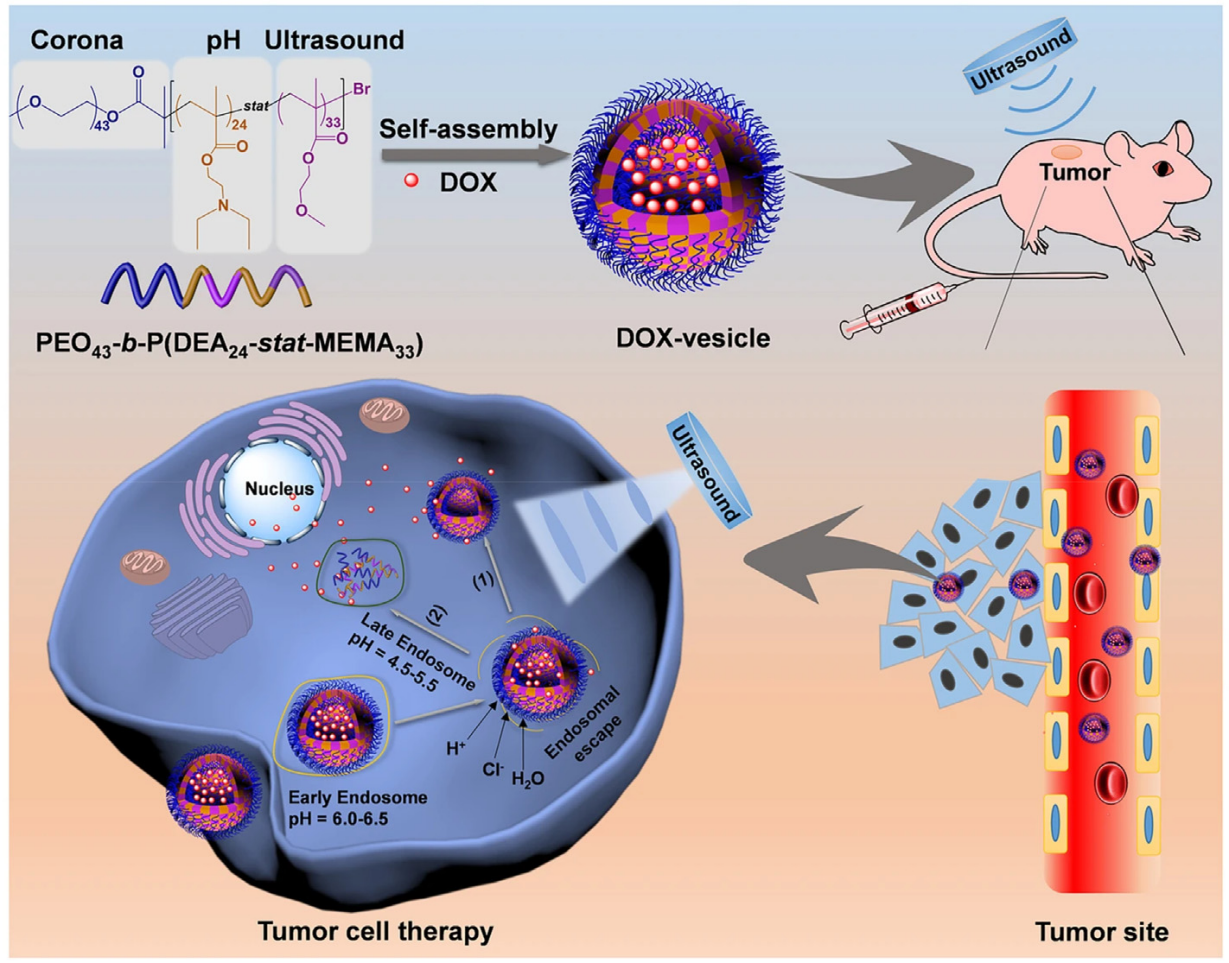
Schematic illustration of ultrasound-sensitive targeted Doxorubicin drug delivery in tumor cells. Copyright © 2020, ELSEVIER Publishing Group. Replicated with permission from Ref. [214].

US-responsive hydrogels have presented outstanding promises for diverse biomedical intentions as drug delivery and tissue engineering. They can boost UScontrast, support image-guided, and provide US-responsive precise release of drugs [206]. A cross-linked US-sensitive hydrogel system has been designed by Huebsch et al., for self-healing and drug delivery exploitations [202]. The US could trigger reversible disruption of calcium crosslinked Alg. hydrogels, afterward, they could undergo self-healing. Furthermore, this system could provide different dose releases of therapeutics depending on the status of the US trigger (on/off) over multiple cycles. For repeated drug delivery using remote US trigger, several attempts were performed to influence the discharge of premature therapeutics from the gel or liposomes [207], a blend of drug carriers [208], and micro bubbles (MBs) [209] added to the reaction mixture of the in-situ gel. The US-responsive nanocarriers blended with a coupling gel could allow the transdermal delivery of some macromolecules like diclofenac Ins. (MW: 6000), erythropoietin (MW: 48 ​000), interferon-γ (MW: 17 ​000), or ovalbumin (MW: 43 ​000) as model vaccines through murine skin [116,188,210,211]. Alg. hydrogels were formulated as US-responsive tailored chemotherapeutics delivery systems [208]. Moreover, Kennedy and co-workers have successfully utilized this system in tissue engineering [212]. Furthermore, US-responsive hydrogels were fabricated for tailored chemotherapy delivery [213,214] as illustrated in Fig. 5, and self-healing purposes [202]. Xu F. et al. [215], fabricated acoustic assembled PEG-based hydrogels. The developed microgels exhibited outstanding compatibility with the encapsulated cells. The acoustic assembly technique provided a facile, cost-effective, quick, and non-invasive tool with potential promises for assembly of intricate 3D cell platforms as constructs for monitoring of interactions between cells and biomaterials, as well as cell response to different drugs.

Special nanoparticles were exploited as US contrast agents to design US imageable tissue engineering platforms. In that context, ZnO nanoparticles were incorporated in poly(N, N-dimethyl acrylamide-co-methacrylic acid) hydrogels to afford a remote post-implantation periodic assessment of organ deformity [216].

#### 3.2.2 Chemical-responsive hydrogels

##### 3.2.2.1 PH-responsive hydrogels

Based on the variable pH of the adjacent environment, pH-responsive hydrogels exhibit responsive swelling in water attributed to their hydrophobic moiety. PAA, poly(methacrylic acid) (PMAA), PAAm, poly(dimethyl aminoethyl methacrylate) (PDMAEMA), poly(diethyl aminoethyl methacrylate) (PDEAEMA), and their copolymers, represent the top leading and conventional pH-sensitive synthetic polymers utilized to design pH-responsive hydrogel systems [96]. Several naturally occurring polymers as Alg., CH, gelatin, and albumin also have pH-responsive swelling capacity. PH-responsive polymeric platforms are usually prepared using polymers developed by ionizable functional groups as –COOH, –OH, –NH_2_, –N<, –CONH_2_, and –SO_3_H [100,186,217]. Upon disclosure to a hydrous solution (solvent) with definite ionic power and pH, these functional groups are ionized with stable charges that display electric repulsion with subsequent swelling-deswelling behavior based on the pH values of the media [100]. Hydrogels containing cationic groups as PDMAEMA, CH, and PDEAEMA swell at pH lower than pKa and deswell at pH higher than pKa [100,218,219]. To synthesize pH-sensitive hydrogels, several natural and artificial polymers with ionizable pendant entities can be exploited [100]. Multiple techniques are adopted to synthesize pH-sensitive hydrogels including FRP chemical [220] and physical [221] crosslinking, injection emulsion polymerization [20], grafted polymerization [222], radiation polymerization and crosslinking methods [223], covalent bondings [224,225], template polymerization [226], and click reactions [227]. Depending on free radical in-situ monomers polymerization; methacrylate and AAm using MBAAm chemical crosslinking compound. Hibbins and co-workers synthesized a novel pH-responsive hydrogel employed for drug distribution [220]. Shantha and Harding exploited the same polymerization and crosslinking approaches using polyethylene glycol diacrylate (PEGDA), N-vinylpyrrolidone (NVP), and CH to produce pH-sensitive hydrogels. The –NH_2_ groups of CH could ionize under acidic conditions (pH 1.2). In another elegant work Yuk, H. et al. [228], fabricated outstanding wearable skin-inspired devices that merge PH-responsive hydrogels, elastomers, electric circuits, and microfluidic channels. Their approach was based on three distinctive steps; first, prior to bonding, the hydrogels and elastomers were pre-shaped to preserve their entire structure. Then, chemical bonding of hydrogels with cured elastomers after their amendment with benzophenone. Eventually, the creation of robust surfaces using dissipative capabilities tough hydrogels (PAAm-Alg., PAAm-hyaluronan, PAAm-CH, PEGDA-Alg., and PEGDA-hyaluronan). These robust hybrid systems merged the merits of the hydrogels and the elastomers. Hence, they fit diverse applications as hybrids against dehydration, circuit boards of elastic hydrogels designed on elastomer, and interactive and stretchable hybrid microfluidics.

Dual stimuli-responsive hydrogels can be produced via the blending of these pH-responsive polysaccharides with thermo-sensitive polymers. These unique mix polymers can react to the alteration of both pH and/or temperature inside the body [1]. The graft polymerization method was utilized in combination with polymerized free radical chemical crosslinking to produce both pH and thermo dual sensitive hydrogels relied on the grafting of cellulose nano-whiskers on AAm (CNWs/AAm). To attain the ultimate hydrogel, MBAAm crosslinkers were utilized to crosslink the CNWs/AAm polymerized with NIPAAm [222]. Suhag and co-workers have adopted the FRP approach to develop pH-sensitive polymeric hydrogels through the physical interplay between 2-DMAEMA polymers and AAc [221].

The utility of pH-sensitive hydrogels in different biomedical applications has a promising potential, mostly in the transport and integration of biomolecules. They were frequently designed as controlled oral drug release systems and utilized as targeted Ins. delivery vehicles in clinical purposes [229,230]. The usage of pH-responsive hydrogels for site-specific proteinous drug delivery was studied using a gel polymeric carrier consisting of a water-soluble CH-based derivative (N, O-carboxymethyl chitosan (NOCC)) and Alg. hydrocolloid cross-linked by a fruit extract (genipin). Out of this study, NOCC/Alg. hydrogel cross-linked with genipin has presented encouraging results as a successful targeted proteinous drug delivery device [231]. Rao et al., have produced a pH-responsive cellulose nanocrystal-enriched poly(acrylamidoglycolic acid) nanocomposite through ionization of hydrogel carboxylic acid (R–COOH) moieties. This cytocompatible hydrogel system was successfully employed as a pH-sensitive diclofenac drug release system [229]. The initial release rate that occurred under acidic conditions (pH ​= ​1.2) was low, while the maximum release rate occurred under physiological conditions (pH ​= ​7.4). These nanocomposite hydrogel platforms, with their power to over gastric acidic conditions, present a promising potential as orally administrable post-gastric drug-releasing systems.

Recently, Wu et al., have designed injectable hypersensitive pH-responsive hydrogels intended for self-healing [232]. Other researchers have produced interesting pH-responsive HA/poly-l-lysine hydrogels enriched with mesenchymal stem cell-derived exosomes that could offer multifunctional biomolecules to achieve augmented wound healing, and prompt angiogenesis with re-epithelization of skin injuries [233]. Lee Y. et al. [234], designed outstanding 3D printed magnetic, temperature, pH, and cationic multi-responsive hydrogels (PNIPAAM-AAc). They served successfully as actuators with potency for tailored occlusion of capillaries. Current progress in the pH-sensitive hydrogels paves the way for future uses of pluri-functional microrobots produced in 3D and 4D for lab and organ-on-a-chip operations, as well as finely targeted obstructive therapies.

##### 3.2.2.2 Glucose-responsive hydrogels

Glucose-responsive hydrogels could be employed in different forms including, but not restricted to, microgels, nanogels, vesicles, micelles, and mesoporous nanoparticles [235]. Covalent bonding is amongst the most prevalent methods to synthesize glucose-responsive hydrogels. Yang T. et al., have blended poly(ethylene oxide)-b-poly vinyl alcohol (PEO-b-PVA) diblock polymer, α-CD, and phenylboronic acid (PBA)-ended PEO crosslinker to synthesize a glucose-sensitive hydrogel. The covalent linkage between PBA and PVA could provide glucose-sensitive crosslinking. While the implication of PEO and α-CD could enhance hydrogel production and improve its stability. When disclosed to sugar, the hydrogel platform dissolves with a concurrent targeted discharge of the laden protein drugs [236]. In another elegant investigation, glucose-responsive polymeric hydrogels have been developed through the FRP stepwise process. Firstly, an AAm and MBAAm in 4-(2-hydroxyethyl) piperazine-1-ethane sulfonic acid (HEPES) stock solution was prepared. Then, N,N,N′,N′-tetramethylethylenediamine (TEMED), and N-(3-dimethyl aminopropyl acrylamide) (DMAPAAm) were blended with the solution. The free radical crosslinking was propagated using a 3-acrylamidophenylboronate (3-APB) initiator. Eventually, the formed glucose-responsive hydrogel was kept in a porous sheath and presented substantial transition in response to the alteration of the adjacent glucose levels [237,238].

The growing demand towards improving the monitoring of blood glucose levels in diabetics has rendered this field more promising for further scientific investigations. Glucose-responsive hydrogels were introduced as outstanding Ins. release system intended to perform as an industrial alternative to the damaged pancreas, that can release a precise dose of Ins. in correlation with the blood glucose level [239,240].

Generally, glucose-sensitive hydrogels are categorized into three main classes: glucose oxidase (GOx) hydrogels, concanavalin A (Con A) hydrogels, and phenylboronic acid (PBA) hydrogels [241]. The principle of the most adopted approach to the design of auto-regulated glucose-responsive Ins. release devices is the entrapping of enzyme (catalase and GOx) nanoparticles into a pH-sensitive hydrogel involving an Ins. saturated solution. Under hyperglycemic conditions, the enzymatic transformation of glucose into gluconic acid undergoes resulting in hydrogel expansion and widening of mesh network pores with a subsequent discharge of Ins. outside the hydrogel matrix. Once the Ins. is released, the blood glucose level drops, and consequently the pH increase resulting in the cessation of further Ins. release [[242], [243], [244], [245]]. The glucose-responsive hydrogel system works as a self-activating valve attuned to discharge Ins. at low release rates in normoglycemic states and the rates increase in hyperglycemic circumstances [242].

Several successful hybrid glucose-responsive platforms have been established with Ins. delivery properties [[246], [247], [248]]. Gu et al. have designed uniform, hybrid hydrogels for regulated glucose-responsive delivery of Ins. in a hyperglycemic mouse model of type I diabetes. The system comprised of a pH-responsive CH matrix, glucose-specific enzymes encapsulated into nano-capsules, and recombinant human Ins. and succeeded to attain responsive Ins. release and decrease the level of blood glucose [242]. This elegant work has paved the way to design self-controlled glucose-sensitive hybrid platforms.

However, being highly promising, the fact that most glucose-responsive nanocomposite hydrogels are enzyme-rich makes their implementation in clinical applications challenging owing to cost concerns, enzymatic stability, and potential immune responses because of the administration of enzyme-dependent constructs [249,250].

The utilization of biomimetic glucose-binding moieties (i.e., boronic acids) provides cost-effective, commercially available products combined with amended glucose-binding capacities over an extensive range of environmental circumstances (i.e., temperature, humidity, and the existence of interfering compounds) [251,252]. Recently, research endeavors have additionally reinforced the multifunctional capacities of boronic-based scaffolds both in microneedle-based hydrogel matrices and isolated nanocarriers to treat diabetes [253,254]. Likewise, in the focus of nanocomposite glucose-responsive hydrogels, maltose- and boronate-functionalized heparin biomaterials were merged with heparin-based nanospheres bearing maltose functional groups to yield 3D hybrid platforms [255]. These platforms were further improved via loading of Ins.-like growth factor-1 (IGF-1) in the constituents of the nanocarrier. Moreover, they presented successful cytocompatible entrapping and propagation of human adipose-derived stem cells.

**Fig. 6:**
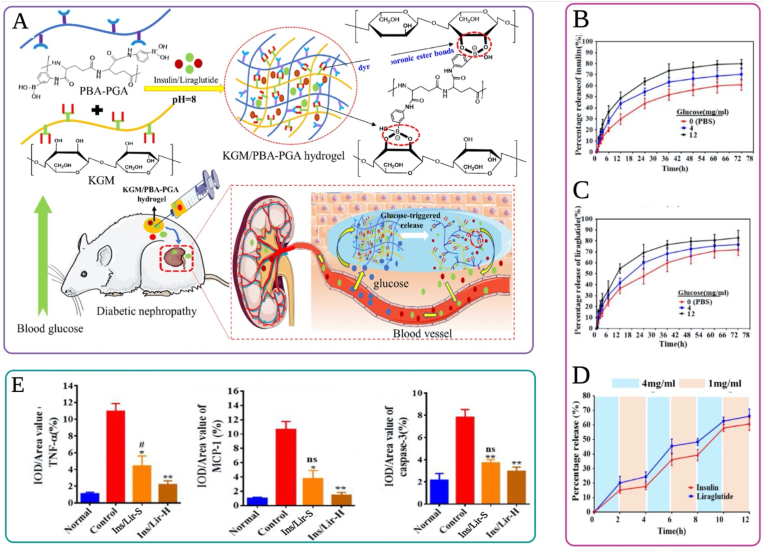
Schematic illustration of injectable glucose-responsive (KGM/PBA-PGA) hydrogel for co-delivery of insulin (Ins.) and liraglutide (Lir.) to prevent early diabetic nephropathy (DN) in rats. Glucose sensitivity of KGM/PBA-PGA hydrogels after exposure to different glucose levels (A). Glucose-responsive release of Ins. (B), Ins. and Lir. (C) from KGM/PBA-PGA hydrogel in PBS containing different glucose levels at pH 7.4, and 37 ​°C. On/off regulated glucose-responsive release profile of Ins. and Lir. from the KGM/PBA-PGA hydrogel as a function of glucose concentrations (1 ​mg/mL and 4 ​mg/mL) (P ∗ ​< ​0.05; ∗∗*P* ​< ​0.01, *n* ​= ​3) (D). As a result, there was a suppression of the inflammation with a reduction of the expression of the inflammatory markers (TNF-α, MCP-1, and caspase-3) (E). Copyright © 2021, ELSEVIER Publishing Group. Replicated with permission from Ref. [248].

Recently, Meng-Qi Tong and coworkers, based on dynamic boronic esters bonds between konjac glucomannan (KGM) and phenylboronic acid-grafted *γ*-Polyglutamic acid (PBA-PGA) could design a glucose-responsive (KGM/PBA-PGA) hydrogel as cargo for codelivery of Ins. and liraglutide (Lir.) to control diabetic nephropathy (DN) [248] as demonstrated in Fig. 6A. KGM/PBA-PGA hydrogel system has presented a substantial capacity to provide glucose-responsive release of Ins and Lir. in PBS containing different glucose levels at 37 ​°C and 7.4 pH (Fig. 6B and C). Moreover, it exhibited a pulsatile Ins. and Lir. release under different normo- (1 ​mg/mL) and hyper-glycaemic (4 ​mg/mL) conditions. In response to different glucose levels, it could also reverse its Ins. or Lir. release behavior among 3 successive cycles (Fig. 6D). Furthermore, the in vivo results on diabetic rats have confirmed the high capacity KGM/PBA-PGA hydrogel for dual release of Ins. and Lir. that shared effectively to provoke the anti-inflammation effect via inhibition of TNF- *α* and MCP-1 expression (Fig. 6E) and suppress cellular apoptosis.

##### 3.2.2.3 Ionic strength and redox-responsive hydrogels

The ionization of the polymeric network is the main factor that determines the extent of its responsive swelling. The equilibrium and swelling behavior dynamic are controlled by numerous factors as the ionic power and pH of the swelling ambiance, and level of ionization [256]. The pH-responsive hydrogel swelling is the consequence of the interplay between the pH and the ionic power of the liquid surrounding the hydrogel [257]. The existence of monovalent and bivalent positive ions as Na^+^, K^+^, Mg^2+^, and Ca^2+^ in the tears of the eye makes the ion-triggered gelling devices a potential ocular drug conveying system [258] as they can be converted from the aqueous form to a diaphanous gel in the existence of these cations [259]. Ionic cross-linking is a facile approach to prepare successful hydrogel polymers implemented for different biomedical applications. Ionically crosslinked CH/gelatin hydrogels are appealing for chemical-responsive platforms with implementations as biosensors and tailored release devices [260]. In a similar context, ionically crosslinked CH hydrogels are recognized to be well-tolerated and biocompatible. Hence, they were extensively utilized in potential biomedical [261], pharmaceutical [262], and biosensing [263] applications.

Reduction–oxidation (redox) potency is a biological factor that is affected by many circumstances and can be changed under some diseased conditions, like inflammation, cancer, or hypoxic states [264,265]. Redox potential is recognized, similar to pH gradients that exist within the endocytic components of live cells, via cytosolic and intracellular organelles high glutathione (GSH) concentrations compared to the extracellular surroundings that are inimitably utilized to trigger intracellular drugs [266].

Redox-responsive hydrogels were developed to recognize reactive oxygen species (ROS) that consequently protect cells from ROS-induced oxidation. ROS are very reactive agents released from cell metabolism and are abundant in the human body. Moreover, they provide a crucial contribution to cell signaling despite their potent oxidation-induced destructive effect on cellular fluids, nucleic acids, and proteins [267,268]. Consequently, progressive production of ROS is usually associated with many systemic disorders and disturbance of several functions of the body [269,270]. Disclosure of the redox-sensitive hydrogels to an oxidant like NaClO, their viscosity diminishes and present prompt solution formation. In the contrast, their viscosity raises and present prompt gel formation when exposed to reducing agents as GSH. Likely, the same redox-responsive behavior was encountered upon exposure to electrochemical prompted redox stimulation [269,271].

To synthesize redox-responsive hydrogels, redox-responsive structures were integrated within a thermo-sensitive media. It was reported that a thiol-yne reaction was utilized to functionalize a polymer developed through the ring-opening polymerization method to support the redox-responsive capacity to the hydrogel polymer. The produced polymer could exhibit redox-triggered responsiveness to the chemical stimulation by H_2_O_2_ [269]. Redox-responsive organo-metallic structures could be also integrated to produce redox-responsive hydrogels. Nakahata and co-workers could develop redox-responsive hydrogels utilizing host-guest polymeric interplays via incorporation of redox-responsive ferrocene (FE) and PAAc (PAAc/FE) as a guest polymer to PAAc/β-CD host polymer [271]. Furthermore, disulfide linkage-dependent crosslinking could provide a frequently utilized alternative technique to produce redox-responsive hydrogels via thiol-disulfide interchange reactivity [19].

Liang and Kiick have designed redox-responsive PEG-based polymeric hydrogels employed for drug-laden liposome delivery in thiol-rich environments [272]. This work had presented a simple way to fabricate improved, hybrid drug delivery cargos that display unique chemical degradation.

The fact that ROS, which is highly involved in the pathological exacerbation of the affected sites, are abundant in the highly inflamed/ischemic areas of the tissues or regions with infection foci has inspired researchers to design ROS-responsive hydrogels for enhancement of wound healing, inhibition/control of pathogen growth, and anti-inflammatory therapeutic applications [273]. Moreover, for further improvement of anti-infection therapeutic strategies, ROS-responsive (PAAg-PGFe) nanocomposite hydrogel systems made of polyacrylic acid (PA) based hydrogels coated with silver (Ag) nanoparticles and iron (Fe2+/Fe3+)-contained polyglutamic acid (PG) networks have presented substantial anti-infection capacity with improved wound healing [274].

In another elegant work, researchers have fabricated ROS-responsive hydrogels utilized as an injectable wound dressing. These unique networks based on PEG, sodium Alg., and pectin have exhibited outstanding anti-inflammatory peptide release with extremely enhanced wound healing abilities in a full-thickness wound mouse model [275].

#### 3.2.3 Biological-responsive hydrogels

##### 3.2.3.1 Enzyme-responsive hydrogels

It is an interesting concept that the biomolecules and peptide sequences can be cleaved by certain enzymes that crosslink the hydrogels [276]. The catalytic behaviors of these enzymes on the substrate can result in alterations in the gel swelling behavior [3]. Matrix metalloproteinases (MMPs) families, with their high ability to break ECM molecules, come at the forefront of enzymes broadly employed for tissue engineering and regeneration [277,278]. They were highly engaged in tumor invasion and implicated in tissue remodeling processes [17].

To synthesize new enzyme-responsive hydrogels, enzymes that serve as natural triggering agents and enzyme-catalyzed responses were the potential activating compounds utilized [[279], [280], [281]]. As biomolecules, the enzymatic release profile can be managed through adjustment of the hydrogel polymer physical traits of the and tunning their response [282]. Enzyme-sensitive hydrogel systems with low molecular weight gelators (LMWG) were presented as modern expansions in this sector where the enzymes can adapt and distinguish the hydrogel surface [283,284]. To synthesize enzyme-responsive hydrogels intended mainly for implementations in the biomedicine, enzyme-catalyzed approach where the enzyme serves as a catalyst able to cause morphologic and/or chemical alterations in the hydrogels is one of the chief methods for this intention. These hydrogels should contain incorporated enzyme detecting components as linkers that are necessary to be available to the enzymes to secure enzyme-catalyzed reactivity. The rection between the enzyme and the linker induces responsive chemical and/or physical modifications in the smart hydrogel matrix including degeneration or morphologic phase transitions [282,285,286].

Some enzyme-sensitive hydrogels present responsive dissolution of the hydrogel platform to the enzymatic stimuli. These smart/stimuli-responsive hydrogels can be developed via one of two methods: the first technique is the simple covalent crosslinking of the hydrogel polymer. The second one is the establishment of supramolecular construction via numerous self-assembling particles (hydrogelators) that intermingle to produce nanofibers that intermingle to produce the hydrogel [284,287,288].

Hydrogel chemical synthesis is usually achieved through covalent crosslinking of the polymers. They are assembled through two main procedures; the biodegradable materials are dissolved. The physical hydrogels are produced via physical crosslinking of the biological elements [116,289,290]. The enzyme reactive entities present in the center or the side chain of the enzyme-responsive hydrogels as unstable bondings that trigger enzyme-responsive alterations and conversion in the organization of the hydrogel polymer through many non-covalent reactions [116,281].

**Fig. 7:**
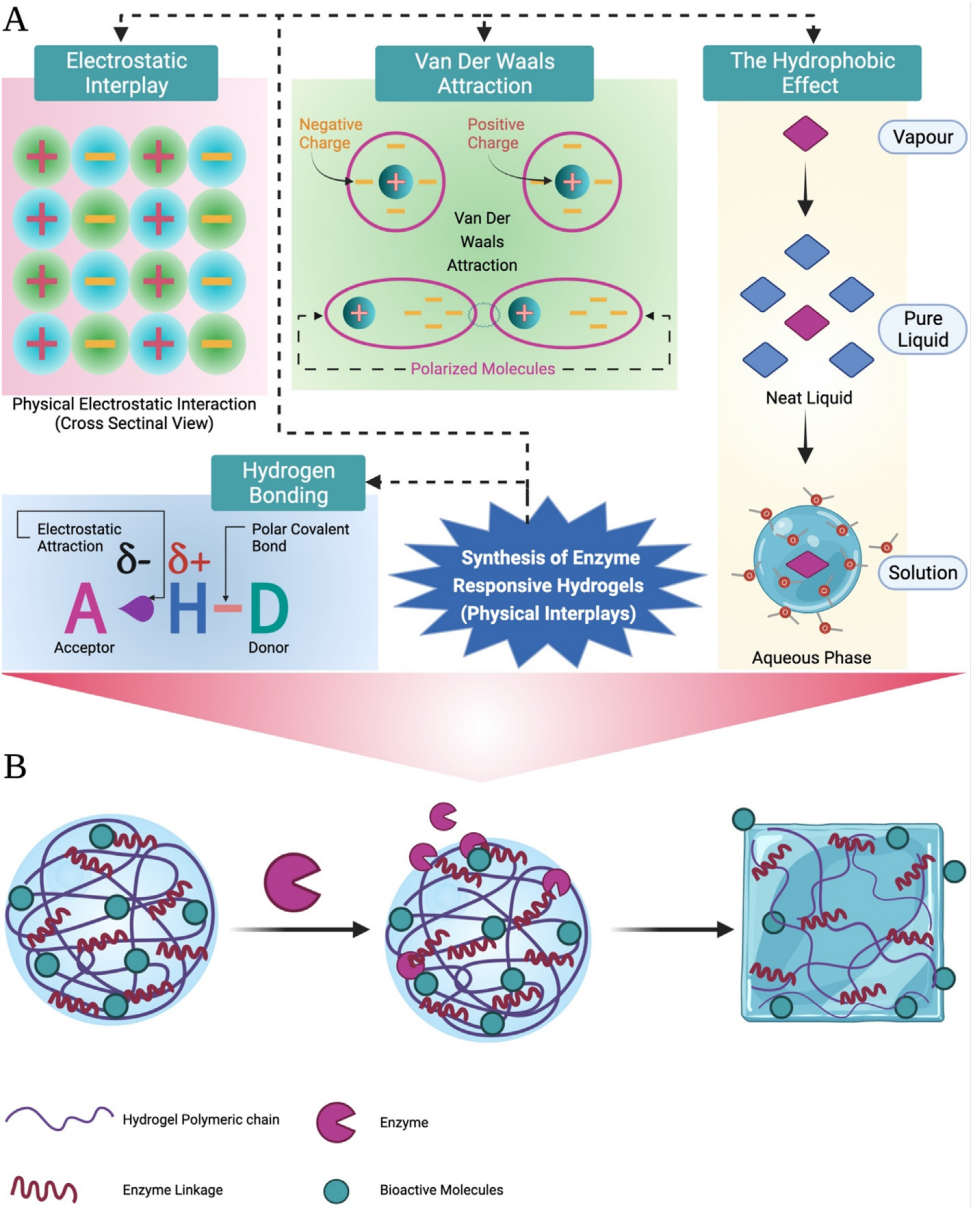
Schematic illustration of enzyme-responsive hydrogels synthesis and their enzyme triggered response. (A) Physical interplays to synthesize enzyme-responsive hydrogels. (B) Mechanism of hydrogel response to an enzyme with the subsequent enzyme-responsive release of bioactive molecules.

The molecular interplays in the form of formation of several multiple chemical bondings like electrostatic interplays, hydrophobic interplays, hydrogen bonds, van der Waals reactions, π-π interplays, or any further mixing of these bonds (Fig. 7A) induce alteration of hydrogels shell characters including self-assembly, supramolecular constructions, and swelling/deswelling behaviors [291] as illustrated in Fig. 7B. Recently, Maki T. et al. could synthesized an enzyme-responsive urea-based hydrogel through the integration of rhodamine 6G (RH6G) and urea in the hydrogel structure. The produced hydrogel could exhibit substantial enzyme-sensitive sol-gel phase transition in the existence of β-galactosidase (β-Gal) enzyme [292]. In another study, Shen X. et al., could develop a hydrogel via covalently crosslinking of chitin and β-(1 ​→ ​4) connected _d_-glucose unit that could be integrated inside the hydrogel and utilized for tailored 2-acetamido-2-deoxy-β- _d_ -glucose-responsive drug delivery [116].

Recently, interesting MMP-degradable HA hydrogels were fabricated to function as cargos for the liberation of loaded vascular endothelial growth factor (VEGF) and platelet-derived growth factor-BB (PDGF-BB) to the site of injury [293]. Furthermore, it was likely to successfully exhibit consecutive release of VEGF and PDGF-BB in this special order to improve tissue healing in ischemic wounds, compared to the reverse order or uncontrolled growth factor release [294].

Enzymes show high specific responsiveness and incorporation in different biological processes [295,296]. Thus, enzyme-responsive materials can be synthesized to provide specific responses through the integration of specific substrates. Through catalyzing or inducing physiological reactions for tissue engineering, and drug delivery devices, enzymes play a vital role in most biomedical aspects [1,297,298]. Enzyme-sensitive hydrogels are frequently employed as efficient drug delivery platforms to the colon where the proteolytic activity is lesser than that in the small intestines [299,300]. A diversity of hydrogels, mainly enzyme-sensitive hydrogels, are being deemed and tailored as colon-targeted drug delivery [301]. Aimetti et al., have designed a smart hydrogel system intended for drug release and merged various kinds of smart/stimuli-responsive hydrogels like enzyme-sensitive hydrogel nanoparticles, drug-responsive hydrogels, magnetic-sensitive hydrogels, and hydrogels for dual protein carriage [302]. One benefit of enzyme-responsive hydrogels, attributed to their mild swelling capacity at a small pH value, is their capacity to escape the digestion of proteinous drugs by the gastric proteolytic enzymes [303]. Nanocrystalline chitin films fabricated by Wang and Esker for enzyme control have been declared as an enzyme-sensitive moiety [304]. Similarly, the CH hydrogel was amended with a fluorogenic substrate and utilized as an enzyme-sensing hydrogel technique [305].

Enzyme-responsive hydrogels could be attained either through employing intrinsically sensitive natural polymers (i.e., HA, gelatin, collagen, fibrin) or amendment of the non-recognizable biomaterials could use enzyme-responsive linkers [45]. HA-based enzyme-responsive hydrogels showed satisfactory potentials for applications in biomedicine. That is because HA is a substrate to the Hyaluronidase enzyme that presents overexpression in invasive malignant tumors and is discharged by the pathogenic microbes at sites of infection [17,306].

Currently, bioinstructive nucleic acids, either naked plasmid DNA (pDNA) or pDNA-loaded nanoparticles, delivery to program cell activities is an elegant technique substitute to the release of growth factors [1]. The main restriction of this approach is the affinity of DNA polyplexes to aggregate in HA or fibrin-based hydrogels that affects the efficacy of gene therapeutics loading in the hydrogel matrices. To fix this problem, intense non-aggregated pDNA nanoparticles were encapsulated within enzyme-degradable acrylated HA or fibrin-based hydrogel matrices [307]. In another report, to potentiate gene-based bioactive signal delivery to direct stem cell behavior and regenerate diabetic ulcerative wounds, researchers could design soft HA-based hydrogel scaffolds fabricate with MMP-degradable linkers and loaded with pDNA nanoparticles [308,309].

Clustered regularly interspaced short palindromic repeats (CRISPR) and CRISPR-associated nuclease effectors (Cas)), promising gene-editing means, have also lately set proof-of-concept presentations for production of nuclease-sensitive hydrogels to broaden the versatility of enzyme-responsive hydrogel systems that exhibit programmable delivery of live cells, and biomolecules [310].

##### 3.2.3.2 Antigen/antibody-responsive hydrogels

The basis of working of antigen/antibody-responsive hydrogels relies on the detection of antibodies or antigen halted on a transducer to antigen or antibodies. The strength of the produced signals is proportionate to the analytes’ concentration [189]. The antigens or the antibodies can be entangled inside the hydrogel matrix through either chemical conjugation of them in the hydrogel or copolymerization between their binding fragment and the hydrogel [311,312]. Hydrogel matrices were employed chiefly to encapsulate these analytes thanks to their exceptional properties as porous configuration, elevated binding capacity, and high chemical stability [313].

To synthesize antigen-responsive hydrogels for biosensing applications, many methods were utilized, among them are the following: antigen incorporation in the hydrogel, antigen chemical coupling, and the exploitation of antigen-antibody reaction as a double-sided cross-linker [24,311]. It was potential to produce antigen-sensitive hydrogels from supramolecular hydrogels; the supramolecular hydrogel was fabricated through the employment of the self-assembly via manipulation of LMWG. In the cell culture media, the steadiness of supramolecular hydrogels.

Permits their additional advancement to cell and enzyme-sensitive hydrogels with unique fluorescent drug release mechanisms via catalyzed proteolysis of specific prostate antigens. This was a very promising and efficient way to diagnose prostate cancer [25,314,315]. Zhang and co-investigators have synthesized a smart antigen-responsive hydrogel as follows: the dextran was grafted in an antigen (fluorescein isothiocyanate (FITC)) and an antibody (sheep anti-FITC IgG). The FITC and IgG antigen-antibody reaction functioned to confirm further hydrogel polymeric stability [316]. In another work by Yang H. et al., they could, through polymerization of NIPAAm and IgG2a via redox propagation and MBAAm-mediated chemical crosslinking, synthesize a hydrogel [24]. A target-sensitive hydrogel was fabricated utilizing an analogous mechanism of amendment in the density of their crosslinking responsive to the target compounds [26].

Recently, antigen-responsive hydrogels synthesized through DNA grafted PAAm and DNA cross-linking for target perception were efficient in the precise detection of toxins and drugs as lead ions, cocaine, and ochratoxin A with high sensitivity. The hydrogels were synthesized through the hybridization of the linker DNA (DNAzyme or aptamer) and the polymeric elements. The preloaded nanoparticles are discharged outside the hydrogel matrices responsive to the target molecules. They possess suitable stability and outstanding catalytic power to decompose H_2_O_2_ and liberation of O_2_ which produce significant raise of the pressure that can be promptly traced by tension meter [317,318].

Antigen/antibody-responsive hydrogels present a very promising potential regarding the fabrication of novel smart sensors [189]. Certain natural polymers, mainly CH, were verified to boost the immunogenicity of antigens with weak immune reactions in the form of liquid and micro/nanoparticles [319]. Alg.-coated CH microparticles have been fabricated by Li et al., as oral antigen carriers to fit the requisite for mucosal vaccines [320].

Randriantsilefisoa et al. provided a significant example in 2019, they could synthesize a soft and robust PEG and dendritic polyglycerol (dPG) hydrogel matrix that was used for precise detection of antibodies [321]. This hydrogel system could covalently interface DYKDDDDK (FLAG®) and GSH peptides for explicitly perceiving their corresponding antibodies. Moreover, Lim et al. have reported a cutting-edge antibody/antigen-based biosensor composed of a bio-conjugated PAAm hydrogel (HBPAAm) that exerts responsive weight change to hepatitis B core antigen (HBcAg) [322]. The affinity crosslinks constructed in the HBPAAm hydrogel network by the precise coupling between the HBcAg and its antibody were arrested but were disrupted in the existence of analyte with induction of free HBcAg concentration-dependent responsive swelling of the hydrogel.

Choi and co-authors have suggested a label-free, specific detection technique for immunoglobulin G (IgG) antibody utilizing hydrogel photonic crystals that is perfect for biomolecular screening due to its capacity to offer a homogenous adjacent water environment [323]. Antigen-sensitive hydrogels could be engaged to produce antigen-sensing devices for biomolecules [322], or drug and protein delivery to the sites of interest [324].

It is preferred, for certain biomedical applications, to possess a material that can present responsive swelling behavior to specific proteins. Souza et al. reported a specific antigen-responsive hydrogel where the antigens are encapsulated in hydrophilic polymer pillars [325]. The hydrogel polymer shrinks as a consequence of an antigen-antibody reaction in the polymer and the lack of free antigen [326]. The prompt recognition and the substantial specificity of antigen/antibody-responsive hydrogels for the detection of antigens/antibodies are not only highly valuable for the early detection of diseases but also the monitoring of the therapeutic efficacy of drugs used for treatment [322]. Collectively, these biological-responsive hydrogels can be incorporated into portable microscale biosensing devices entirely utilized for the point-of-care specimen analysis and real-time prognosis. In the foreseeing future, these smart hydrogel-based bio-sensing systems will provide significant promises for applications in biomedical diagnostics.

## 4 Smart/stimuli-responsive hydrogels employed for different biomedical applications

The capacity to deliver the hydrogels through different either implantation or injection routes (subcutaneous, transdermal, nasal, pulmonary, ocular, vaginal, and rectal routes) has made their use more significant [40]. The distinguishing characteristics of intelligent hydrogel polymers as swelling-deswelling behavior, biocompatibility, hydrophilicity, and biodegradability stand behind their efficacy to fit multiple applications in biomedicine including tissue engineering [275,327,328], targeted delivery [329], biosensors [187,191,192], and actuators [330,331] as presented in Table 3.

**Table 3:** Smart/stimuli-responsive hydrogels employed for different biomedical applications.

Type of smart/stimuli-responsive hydrogel		Smart/stimuli-responsive hydrogel polymer	Smart/stimuli-responsive hydrogel system	Stimuli	Biomedical applications	Year	Refs.
**Physical-responsive hydrogels**	**Temperature-responsive hydrogels**	-Poly(N-isopropylacrylamide) (PNIPAAm)	-PNIPAAM covalently grafted onto a flat substrate that forms the bottom of the microfluidic device	-Temperature	-Microfluidic actuators	2018	[332]
		-Methylacrylate gelatin (GelMA)	-GelMA-PDA-ASP nanocomposite hydrogels	-Temperature	-Dressing to enhance skin wound healing -Targeted delivery of ASP	2021	[333]
		-Polyacrylamide (PAAM) -Poly(N-isopropylacrylamide) (PNIPAAm)	-CNW–PAAm–PNIPAm nanocomposites	-Temperature -PH	-Promising for different biomedical applications	2015	[222]
		-Poly(N-isopropylacrylamide) (PNIPAAm)-Hydrophobically associated polyacrylamide (HAPAM) hydrogel (HAPAM-PNIPAAM)	-Mxene-HAPAM-PNIPAAM nanocomposite double-network hydrogel	-Temperature -Compression/stress	-Smart compression biosensors	2019	[192]
	**Light/photo-responsive hydrogels**	-Polyacrylamide (PAAM)	-Conducting polymer hydrogel (CPH) based on copolymerized PANI and PAM (PAM/PANI CPH)	-NIR light	-Neural tissue engineering	2020	[334]
		-Poly(N-isopropylacrylamide) (PNIPAAM)	-Poly(diketopyrrolopyrrole-alt-3,4 ethylenedioxythiophene)-PNIPAAm	-NIR light	-Targeted and controlled drug delivery for cancer treatment	2017	[136]
		-Poly(N,N-dimethylacrylamide (PDMA)	-PDMA/Coumarin copolymers	-Photo/light	-Promising for different biomedical applications	2018	[125]
	**Electric-responsive hydrogels**	-Carboxymethyl chitosan (CMCH) -Poly(3,4-ethylenedioxythiophene) (PEDOT) conductive polymer layer	-Conductive hydrogels (PEDOT/CMCH)	-Electric field	-Neural Tissue engineering	2018	[335,336]
		-Chitosan (CH)	-PAAM–CH–PPy	-Electric field	-Biosensors -Tissue engineering -Drug Delivery	2018	[149]
	**Magnetic-responsive hydrogels**	-Methylacrylate gelatin (GelMA)	-GelMA-SPIONs nanocomposites	-Magnetic	-Promising for different biomedical applications -3D printing	2020	[170]
		-N-isopropylacrylamide (NIPAAM) -Polyacrylamide (PAAM)	-Nanocomposite hydrogel system (NIPAAm–AAm–PEDGA and MNPs)	-Magnetic	-Microactuators	2016	[174]
		-Polyethylene glycol (PEG)	-Janus microparticle-PEG nanocomposites	-Magnetic	-Microactuators and robotics -3D printing	2021	[173]
	**Pressure/strain-responsive hydrogels**	-Poly(acrylamide-co-Lauryl Methacrylate) (P(AAM-co-LMA))	-Hybrid Latex Nanoparticles (HLPs) crosslinked P(AAM-co-LMA)	-Pressure/strain	-Wearable biosensors	2019	[191]
		-Carboxy Meyhylcellulose (CMC) -Polyacrylamide (PAAM)	-PAAm/CMC semi-interpenetrating network hydrogel	-Pressure/strain	Strain sensors	2020	[190]
	**Ultrasound-responsive hydrogels**	-Alginate (Alg.)	-Ionic crosslinked Alginate hydrogels (Calcium Alginate hydrogels)	-Ultrasound	Drug delivery	2014	[202]
		-Polyethylene Glycol (PEG)	-Sericin/hydrogel scaffold	-Ultrasound	Drug delivery	2019	[199]
**Chemical-responsive hydrogels**	**PH-responsive hydrogels**	-Poly(acrylamidoglycolic acid) (PAGA)	-Poly(acrylamidoglycolic acid) based nanocomposite (PAGA-NC)	-PH	-Drug Delivery	2017	[229]
		-Poly(methacryloyloxyethyl phosphorylcholine-co-4-formylbenzoate ethyl methacrylate) P(MPC-co-FBEMA) copolymers	-P(MPC-co-FBEMA)-ASNP nanocomposite hydrogels	-PH	-Controlled drug delivery	2020	[232]
		-Collagen (Col)	-Col-JK-1	-PH -Enzyme (MMPs)	-Tissue engineering -Drug delivery	2019	[337]
		-Chitosan (CH)	-CH/CG composite	-PH -Salt	Cartilage Tissue Engineering	2018	[338]
		-pre-gel hydrogel solution 80 ​mol% acrylamide, 8 ​mol% 3-acrylamidophenylboronic acid, 10 ​mol% ​*N*-[3-(dimethylamino)propyl]methacrylamide, 2 ​mol% ​*N*,*N*′-methylenebisacrylamide	-Three-layered ​microfluidic sensing device: Bottom: polycarbonate Center: polyvinyl chloride (PVC) Top: another layer of polycarbonate and the smart hydrogel pillars in the microfluidic channels	-PH	-sensors for monitoring disease biomarkers or environmental contaminants in drinking water	2018	[339]
	**Glucose-responsive hydrogels**	-Phenylboronic acid-grafted γ-Polyglutamic acid (PBA-PGA)	-KGM/PBA-PGA	-Glucose	-Drug Delivery	2021	[248]
		-Poly(N-vinylpyrrolidone-co-dimethylamino)ethyl acrylate-co-3-(acrylamido)PBA (P(NVP-co-DMAEA-co-3APBA)) - Ethylene glycol dimethacrylate (EGDMA)	-GR-MN (Glucose-responsive microneedle patch) based on p(NVP-co-DMAEA-co-3APBA)	-Glucose	-Drug Delivery	2020	[254]
	**Ionic strength and Redox-responsive hydrogels**	-Polyethylene Glycol (PEG)	-Poly(ethyleneglycol)-block-poly(γ-propargyl-l-glutamate) (PEG-PPLG)	-Redox -Temperature	-Promising for different biomedical applications	2017	[269]
		-Gelatin -Chitosan (CH)	-Gelatin-chitosan double crosslinked networks	-Ionic strength	-Drug delivery	2011	[260]
**Biological-responsive hydrogels**	**Enzyme-responsive hydrogels**	-Hyaluronic Acid (HA) -Gelatin	-Tyramine conjugated HA (TA-HA) and gelatin	-Enzyme	-Tissue engineering -Drug delivery	2018	[340]
		-Polypeptides (PLys-*b*-(PHIS-*co*-PBLG)-PLys-*b*-(PHIS-*co*-PBLG)-*b*-Plys)	-Pentablock terpolypeptide of the type PLys-*b*-(PHIS-*co*-PBLG)-PLys-*b*-(PHIS-*co*-PBLG)-*b*-PLys	-Enzyme -PH	-Drug delivery	2018	[341]
		-Chitosan (CH)	-Alanyl-alanyl-phenylalanine-7-amido-4-methylcoumarin (AAP-AMC) covalently conjugated to chitosan	-Enzyme	-Biosensors -Tissue engineering	2014	[305]
	**Antigen/antibody-responsive hydrogels**	-Polyacrylamide (PAAM)	- Bio-conjugated polyacrylamide-based hydrogel (HBPAAm hydrogel)	-Antigen	-Biosensors	2020	[322]
		-N-(9-fluorenylmethoxycarbonyl)-L, l-diphenylalanine (Fmoc-FF)	-Peptide hydrogels encapsulating leishmania antigen (N-(9-fluorenylmethoxycarbonyl)-L, l-diphenylalanine (Fmoc-FF) encapsulating leishmania antigen)	-Antigen	-Biosensors	2017	[325]

ASP, Aspirin; CNW, Cellulose nanowiskers; PANI, polyaniline; PPY, polypyrrole; SPIONs, Superparamagnetic iron oxide nanoparticles; ASNP, Silica nanoparticles; CG, Carrageenan; KGM, Konjac glucomannan.

In the former few decades, the field of biodegradable polymeric materials has witnessed substantial progress owing to their excellent properties. The hydrogel employment history in biomedical applications backs to 2017 when Di and et al., designed a transparent hydrogel-based wound dressing composed of bacterial cellulose (BC) and poly(2-hydroxyethyl methacrylate) (PHEMA) hydrogel [328]. Natural polysaccharides derived biomaterials as chitin and CH are appropriate members for biomedical applications being biologically compatible, degradable, and non-toxic materials with antimicrobial activity [144,230,301]. Likewise, Alg. has exhibited an outstanding potential for different biomedical applications with special concern to tissue engineering, drug delivery, wound dressing, and ex vivo cell culture owing to their unique characters and mild gelling circumstances [275,342,343]. BC is a versatile, key biomaterial for upcoming state-of-the-art applications in regenerative medicine especially, tissue engineering and wound reconstruction. They can merge several surface and macromolecular characters, which are crucial for in vivo and ex vivo applications [328].

### 4.1 Smart/stimuli-responsive hydrogels for applications of tissue engineering

The core objective of tissue engineering is to renew the degenerated tissues with the restoration of their functions [4]. Smart/stimuli-responsive hydrogels could express unique characters that made them commonly utilized in tissue engineering applications. They serve as platforms to bridge cell migration to the seat of injury, possess an outstanding capacity to provide conditions that mimic the ECM surrounding environment, could effectively modulate their mechano-physical properties to fit the required application. Besides their great potential as tissue defects repair scaffolds [344]. Stimuli-responsive hydrogels have been consumed for reconstruction of different body tissues, including: cardiac tissue [[345], [346], [347]], neural tissue [335,334], skin [[348], [349], [350]], cornea [143,[351], [352], [353]], bone [[354], [355], [356]], cartilage [[338], [357], [358]], tendon [359,360], meniscus [361], and intervertebral disc [337,362]. Various smart/stimuli-responsive hydrogels employed for the engineering of different tissues are presented in Table 4.

**Table 4:** Smart/stimuli-responsive hydrogels employed for the engineering of different tissues.

Tissue engineering applications	Smart hydrogel polymers	Employed smart hydrogel systems	Stimuli responsiveness	Study model	Purpose of use	Year	Refs.
**Cardiac tissue engineering**	Poly (N-isopropylacrylamide) (PNIPAAm)	PNIPAAm/SWCNTs	Temperature-responsive	-In vivo Study (Rat model of MI)	Delivery of BASCs to the targeted site of MI for myocardial repair	2014	[345]
	Mixed Supramolecular hydrogel of Cur-FFE-SH gelator	Curcumin and NO laden-supramolecular Cur-FFE-ss-ERGD (Cur-FFE-ss-ERGD/Nap-FFGGG-NO)	β -gal enzyme-responsive	-In vivo Study (Mice model of MI)	Dual delivery of curcumin and NO to the targeted site of MI for myocardial repair	2017	[346]
	Poly(vinyl alcohol) (PVA)	PVA-TSPBA	ROS-responsive	-In vivo (rat and pig model of I/R)	Delivery of bFGF to the targeted site of ischemia for myocardial repair	2021	[347]
**Neural tissue engineering**	Carboxymethyl Chitosan (CMCH)	Conductive PEDOT/CMCH hydrogel composites	Electro-responsive	-In vitro (neuron-like rat PC12 ​cells)	Study the cytocompatibility of PEDOT/CMCH for nerve tissue engineering	2018	[335]
	Polyacrylamide (PAM)	Conducting polymer hydrogel (CPH) based on copolymerized PANI and PAM (PAM/PANI CPH)	Light/Photo-responsive	-In vitro (white corded sciatic nerves of a hoptoad) -In Vivo (rat peripheral nerve injury model)	Conductive bridge for replacement of lost peripheral (sciatic) nerve	2020	[334]
**Dermal tissue engineering**	Methacrylate gelatin (GelMA)	GelMA-PDA-ASP nanofibrous hydrogels	Temperature-responsive	-In vitro Study (L929 and HaCaT cells) -In vivo Study (mice skin wound)	Wound dressing for delivery of Aspirin (ASP)	2021	[333]
	Chitosan (CH)	Silver sulfadiazine loaded CH/PVP	PH-responsive	-In vitro Study	Wound dressing for delivery of silver sulfadiazine to the injury site	2019	[349]
	Carboxy Methylcellulose (CMC)	FU-loaded PCNCHFs composites (PCNCHFs@FU)	PH-responsive	-In vitro Study	Wound dressing for delivery of FU to the injury site	2021	[350]
**Corneal tissue engineering**	Chitosan (CH)	CH combined with DGP (CH/DGP)	Temperature-responsive	-In vitro -In vivo (allergic conjunctivitis in New Zealand Albino rabbits and Guinea Pigs)	Ocular drug (levocetirizine dihydrochloride) delivery system	2012	[352]
	Hyaluronic acid (HA)	HA and Pluronic F-127 (HA-F)	Temperature-responsive	-In vitro (BCECs with and without P-PRP)	Regeneration of the injured corneal endothelium	2017	[351]
	Gellan maleate (MA-G)	Gellan maleate (MA-G) with NIPAm (MA-G/NIPAm)	Temperature-responsive	-In vitro -In vivo (rat model)	Ocular drug (Adrenaline and Chloramphenicol) delivery system	2019	[143]
**Osseous tissue engineering**	Chitosan (CH)	Hemicellulose xylan/CH composite	Temperature-responsive	-In vivo (Rat non-union femoral fracture and Mouse tibial fracture)	Enhancement of bone defects repair and regeneration	2016	[356]
	Chitosan (CH)	CH/β-glycerophosphate (CH/β-GP)	Temperature-responsive	-In vivo (Class III bone defects in beagle dogs)	Cargo for delivery of BMP-7 and ORN for periodontal regeneration	2019	[355]
	Alginate (Alg.)	P(Alg-g-NIPAAm) hydrogels mixed with Hydroxyapatite (HAp)	Temperature and Ultrasound-responsive	-In vitro	Cargo for delivery of different therapeutics (NaF, BSA, and BMP-2)	2021	[354]
**Cartilaginous tissue engineering**	Chitosan-g-poly(N-isopropylacrylamide) (CH-g-PNIPAAm)	3D Mesenchymal stem cells (MSCs) encapsulated in CH-g-PNIPAAm (CH-g-PNIPAAm@MSCs)	Temperature-responsive	-In vitro study (Murine MSCs)	Support the proliferation and chondrogenic differentiation of MSCs	2017	[357]
	poly(ε-caprolactone)–poly(ethylene glycol)–poly(ε-caprolactone) (PCEC)	TGF-β1-loaded PCEC	Temperature-responsive	-In vivo (Rat full-thickness knees cartilage defects)	Delivery of TGF-β1 for proper chondrogenesis	2017	[358]
	Chitosan (CH)	CH/CG composites	pH and Ionic strength-responsive	-In vitro Study (ATDC5 Cells)	Support the chondrogenic differentiation of ATDC5 cells	2018	[338]
**Tendinous tissue engineering**	poly(N-isopropylacrylamide) (PNIPAAm)	Hydrophilic biopolymers Chitosan (CH) and Hyaluronic acid (HA) to PNIPAM HA–CH–PNIPAM (HACPN)	Temperature-responsive	-In vitro (NIH 3T3 cells) -In vivo (Rabbit deep flexor tendon)	Enhancement of tendon regeneration with prevention of post-operative peritendinous adhesion	2017	[360]
	Methacrylated ​chondroitin sulfate ​(MA-CS)	Platelet lysate (PL) enriched MA-CS entrapping iron-based superparamagnetic nanoparticles (MA-CS MNPs-PL)	Magnetic- responsive	-In vitro (hTDCs and ​hASCs cells)	Delivery of PL for release of PL-derived growth factors	2018	[359]
**Meniscal tissue engineering**	Glycol Chitosan GC/4-Arm PEG-CHO Hydrogel	BMSC-, and TGF- β1laden crosslinked glycol chitosan (GC) and multialdehyde functionalized 4-arm (4-arm PEG-CHO) (GC/4-Arm PEG-CHO @ BMSC-TGF- β1) hydrogel composites	Temperature-responsive	-In vitro (BMSCs cells) -In vivo (Rabbit meniscal defect)	Release of TGF-β1 to support the fibrochondrogenic differentiation of BMSCs and improve meniscal defects in rabbit model	2020	[361]
**Intervertebral disc tissue engineering**	Polyethylene Glycol (PEG)	miRNA/PGPC Polyplex encapsulated in PEG Hydrogels (miRNA/PGPC@PEG HG)	Enzyme (MMPs)-responsive	-In vivo (Rabbit model)	Two stage miR-29a delivery to the nucleus pulposus cells to promote engineering of degenerated IVD	2018	[362]
	Collagen (Col)	Col-JK1	Dual pH and enzyme-responsive	-In vivo (Rat model of IVDD)	Delivery of hydrogen sulfide (H_2_S) to the site of IVDD	2019	[337]

SWCNTs, Single Wall Carbon Nanotubes; MI, Myocardial Infarction; BASCs, Brown Adipose Tissue Derived Sem Cells, β -gal, β-galactosidase; NO, Nitric Oxide; TSPBA, N1 -(4-boronobenzyl)-N3 - (4-boronophenyl)-N1, N1, N3, N3 -tetramethylpropane-1,3-diaminium; ROS, Reactive Oxygen Species; I/R, Ischemia-Reperfusion; bFGF, Basic fibroblast growth factor; PEDOT, poly(3,4ethylenedioxythiophene); PC12, phaeochromocytoma; CPH, Conducting polymer hydrogel; PANI, polyaniline; β-CD, β-cyclodextrin; NIPAM, N-isopropyl acrylamide; CNT, Carbon nanotubes; PPY, polypyrrole; DAMC, Dialdehyde methylcellulose; PP, polypropylene non-woven fabric; -g-, γ-rays; AA, acrylic acid; PVP, N-vinyl-2-pyrrolidone; CMC, caboxymethyl cellulose; PCNCHFs, Polymer–clay nanocomposite hydrogel films; FU, 5-fluorouracil; BCECs, Bovine corneal endothelial cells; P-PRP, Porcine platelet rich plasma; DGP, disodium α-d-Glucose 1-phosphate; NPC, N-palmitoyl chitosan; NaF, Sodium fluorescein; BSA, Bovine serum albumin; BMP-2, Bone morphogenetic protein 2, BMP-7, Bone morphogenetic protein-7; ORN, ornidazole; CG, Carrageenan; TGF-β1, Transforming growth factor β1; KGN, kartogenin; hTDCs, Human tendon-derived cells; hASCs, Human ​adipose-derived stem cells; ​BMSCs, Bone mesenchymal stromal cells; PGPC, PEG-GPLGVRG-PAsp(DET)-Chole).

#### 4.1.1 Cardiac tissue engineering

Cardiovascular diseases (CVDs) are accounted as one reason for death in the globe; the statistics of people who die due to CVDs are increasing every year [363]. The advancement of cardiac function assessment techniques enables early detection, even prediction of many CVDs [[364], [365], [366], [367]]. Different tissue engineering strategies are employed to overcome this problem considering the limited potency of human cardiac cells to complete regeneration. Various materials have been utilized to repair cardiac and vascular tissues [[368], [369], [370]]. Moreover, many hydrogels have been used to address that limitation. One of them was collagen-fibrin-based hydrogels seeded with human-induced pluripotent stem cells-derived cardiomyocytes (hiPSCs-CM) to regenerate the defective myocardial tissue [371]. This blend of stem cell-laden hydrogel scaffold has revealed a promising potential and played a pivotal role in the engineering of cardiac tissue. Another micro-channeled 3D printed gelatin-based hydrogel system was designed to promote cardiac cell growth and provide the power to utilize stem cells to improve this cardiac regeneration [372]. However, with the significant progress of hydrogel-based systems for cardiac regeneration, further investigations are required to surmount the existing limitations.

**Fig. 8:**
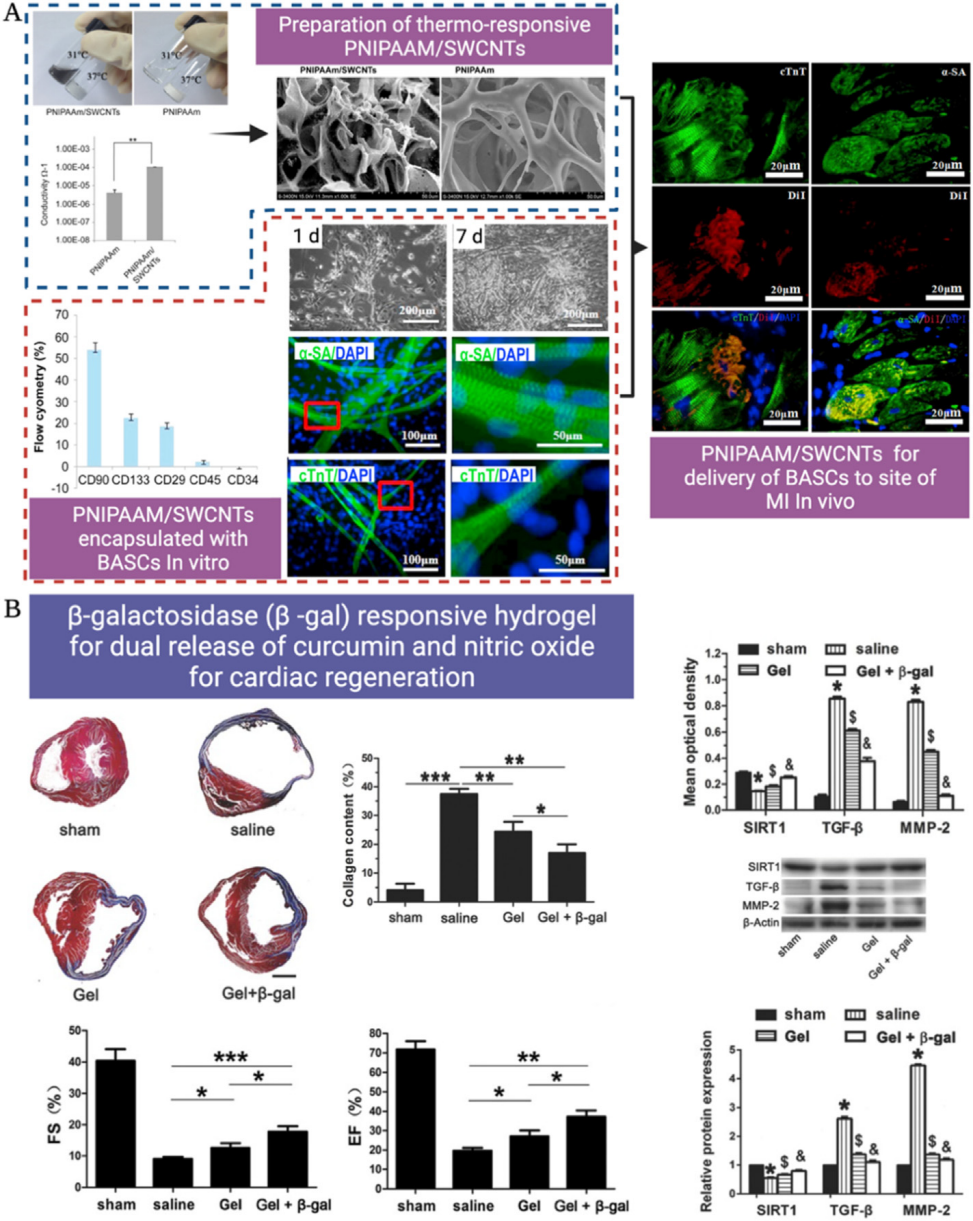
Schematic illustration of smart/stimuli-responsive hydrogels for the engineering of cardiac tissue. (A) PNIPAAm hydrogels blended with single-wall carbon nanotubes (SWCNTs) to form PNIPAAm/SWCNTs composite. Both PNIPAAm/SWCNTs and PNIPAAm hydrogels were temperature-responsive (they liquified below 32 ​°C and gelated at higher temperatures). PNIPAAm/SWCNTs showed significant (∗∗p ​< ​0.01) greater conductivity than PNIPAAm hydrogels. Ex vivo characterization of BASCs showed partial expression of CD90, CD133, CD29, CD45, and CD34. Fluorescent imaging showed differentiation of BASCs towards cardiac lineages. As a result, colocalization of DiI and cardiac proteins (cTnT and α-SA) was observed 4 weeks following in vivo transplantation. Copyright © 2014, ELSEVIER Publishing Group. Replicated with permission from Ref. [345]. (B) Supramolecular β-galactosidase-responsive hydrogels for dual release of NO and curcumin could control the fibrosis and reduce the collagen content evaluated by Masson's trichrome staining. The restoration of heart functions was reflected by improvement of fraction shortening (FS%) and ejection fraction (EF%) post mice model myocardial infarction. The treatment with the hydrogel combined with β-gal (Gel ​+ ​β-gal) has improved the cardiac expression of SIRT1 and reduced the expression of TGF-β1 and MMP-2. Copyright © 2017, The Royal Society of Chemistry. Replicated with permission from Ref. [346].

Smart/stimuli-responsive hydrogels have been shared effectively in cardiac tissue regeneration. Poly (N-isopropylacrylamide) (PNIPAAm) hydrogels have been broadly employed as several therapeutics’ delivery cargoes. However, they presented unsatisfactory bioactivity for encapsulated cells attributed to their limited capacity to support encapsulated cell proliferation [373]. Xia and co-workers have successfully incorporated single-wall carbon nanotubes (SWCNTs) into base PNIPAAm hydrogel to develop a thermo-responsive SWCNTs-modified PNIPAAm (PNIPAAm/SWCNTs) hydrogel with enhanced cytocompatibility [345]. They could assess the bioactivity of the PNIPAAm/SWCNTs hydrogel system to brown adipose-derived stem cells (BASCs), and their efficacy to deliver BASCs to the targeted site of myocardial infarction (MI). PNIPAAm/SWCNTs hydrogels have not only demonstrated significant-high bioactivity to encapsulated BASCs in vitro with enhanced cell proliferation and adhesion, but they also presented a satisfactory capacity to deliver the encapsulated BASCs to the infarct myocardium in vivo with enhanced engraftment of seeded cells at the MI site as illustrated in Fig. 8A. In another research study, Chen et al. have designed a supramolecular hydrogel β-galactosidase (β-gal) enzyme-responsive hydrogel that could release nitric oxide (NO) previously mixed with curcumin to produce a supramolecular hydrogel mixture (Cur-FFE-ss-ERGD).

The combined curcumin and NO therapy could provide marked reduction of collagen deposition, ameliorate the cardiac function, improve the adverse cardiac remodeling, suppress the hypertrophy and apoptosis, attenuate the MMPs expression and transforming growth factor-β1 (TGF-β1), and upregulate the expression of silent information regulator 1 than curcumin alone as illustrated in Fig. 8B [346]. This was attributed to the synergistic action of both molecules and the angiogenesis promotion ability of NO. These attractive results denoted that controllable curcumin and NO codelivery by smart hydrogels might provide a promising alternative for the treatment of CVDs. On the other hand, blood reperfusion to the ischemia tissue produces a large amount of ROS that can aggravate the myocardial damage. Hence, ROS is an imperative marker to diagnose cardiac ischemic/reperfusion (I/R) injuries [374]. In a recent study by Li Z. and coworkers, they could successfully utilize ROS-responsive poly(vinyl alcohol) (PVA) hydrogel system as cargo for ROS triggered delivery of basic fibroblast growth factor (bFGF) to the targeted site of MI [347]. Such a smart hydrogel system has provided a feasible, minimally invasive option for myocardial regeneration with superior angiogenesis and restored heart functions.

#### 4.1.2 Neural tissue engineering

Nerve cells not only present a limited regeneration capacity but a highly complex structure as well. Consequently, the control of the central nervous system (CNS) and other neural damages or injuries is accounted as a significant challenge. In the past, porous hydrogels have been commonly utilized for neural repair owing to the high stability that gives them the aptitude to achieve sustainable tissue growth for long periods. Recently, several in vivo studies utilizing various hydrogel-based scaffolds are conducted to address different yet difficult to treat neurological conditions [375]. Hydrogels have exhibited a substantial capacity for culturing and differentiating neural cells. Also, they have been employed as an efficient release system for neural growth promoters [376,377], antagonists of neural growth inhibitors [378], neurotrophic factors [379,380], and others. Besides, they have been incorporated in neural cell therapy for the provision of localized trophic support and bypass the immune response against the neural cells [381]. Understanding the biological principles of neural regeneration would greatly assist to develop dedicated scaffolds that can closely imitate the physiological requirement of neural regeneration [381]. Hydrogel matrices are emerging a great potential regarding neural tissue engineering, however, that is still in its kickoff. This could be achieved through merging two leading and related issues: (1) fabrication of polymers able to tailor the biological activities and augment the tissue-building capacities, and (2) implementation of cell-based strategies to design hybrid biological constructs or cell-seeded biomaterials.

**Fig. 9:**
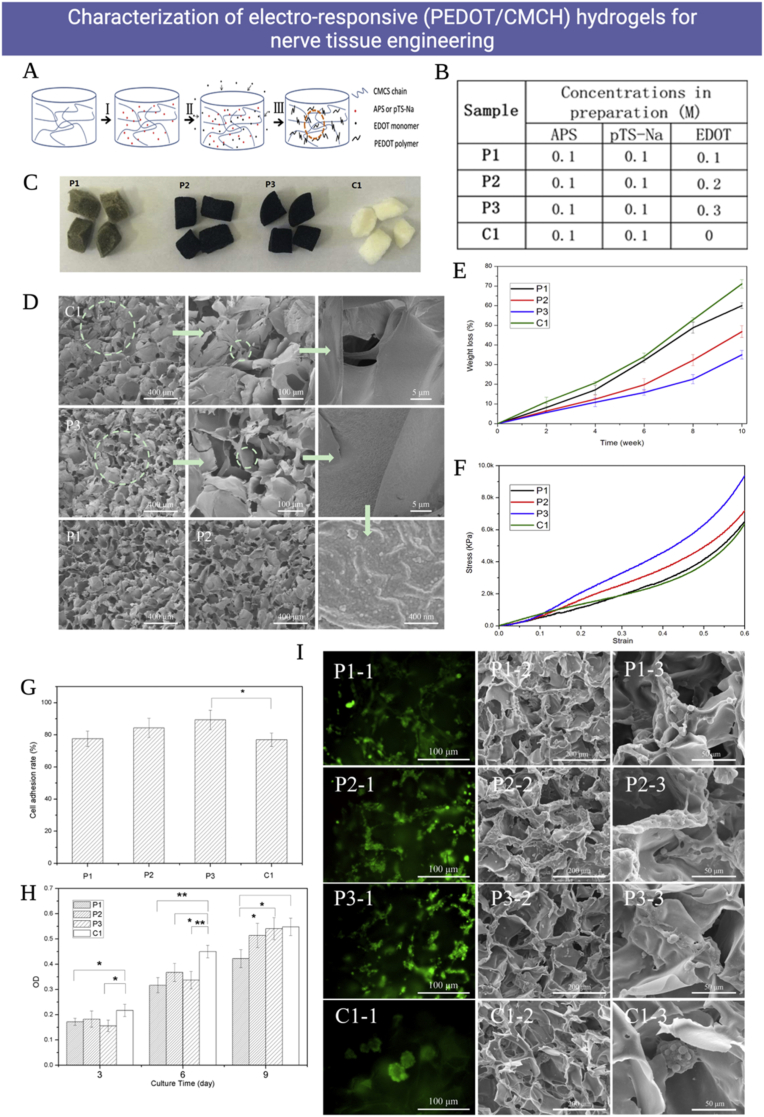
Schematic illustration of electro-responsive (PEDOT/CMCH) for nerve tissue engineering. (A) In-situ polymerization for the formation of electro-conductive (PEDOT/CMCH) hydrogel composites. (B) The table lists different ratios of various monomers and the control sample utilized in that study. (C) The finally prepared conductive PEDOT/CMCH hydrogels. (D) Electron microscopic characterization of CMCH (C1) and PEDOT/CMCH (P1, P2, and P3) hydrogels. (E) Ex vivo degradation (weight loss) profile of CMCH and PEDOT/CMCH different hydrogels. (F) Stress-strain curves for tension-metric characterization of different hydrogels. (G) Cell adhesion profile 4 ​h after incubation. (H) Vitality and multiplication of PC12 ​cells at 3rd, 6th, and 9th days post culture on CMCH and PEDOT/CMCH hydrogels (∗P ​< ​0.05 and ∗∗P ​< ​0.01). (I) Fluorescent microscopic (P1-1–C1-1), SEM (P1-2–C1-2), and magnified SEM (P1-3–C1-3) images of PC12 ​cells adhered to the hydrogels on 3rd day of the incubation. Copyright © 2018, ELSEVIER Publishing Group. Replicated with permission from Ref. [336].

Electro-responsive hydrogels with substantial electromechanical characters played a unique role in biomedical and tissue engineering applications. The electroconductive hydrogels fabricated by Xu C. et al., have been shared successfully in the neuro-regeneration. They designed hydrogel-based conductive composites composed of conductive carboxymethyl chitosan (CMCH) macromolecular network, and conductive poly(3,4ethylenedioxythiophene) (PEDOT) polymeric layer depending on in-situ chemical polymerization [336]. The PEDOT/CMCH hydrogel composites have emerged not only a remarkable ex vivo cytocompatibility with no cytotoxicity to neuron-like rat phaeochromocytoma (PC12) cells but reinforced cell viability, adhesion, and multiplication too. Moreover, the PEDOT conductive components played a fundamental role to improve the biocompatibility, conductivity, and mechanical strain of the CMCH hydrogel matrices as demonstrated in Fig. 9.

Injury of the peripheral nerves, owing to disruption of the transmission of the bioelectric signals between the brain and the portion of the body supplied by the injured nerve, usually results in chronic pain, paralysis, neurological illnesses, or disability in the worst conditions [382]. Recently, Dong et al. have produced a stretchable light-responsive conducting polymer hydrogel (CPH) based on copolymerized polyaniline (PANI) and polyacrylamide (PAM) (PAM/PANI CPH) as a conductive bridge for replacement of lost peripheral (sciatic) nerve [334]. They declared that CPH presented higher conductivity upon illumination with NIR light with enhanced capacity to transmit the bioelectric signals. Moreover, CPH showed substantial ability to adapt to the sudden strain of nerve tissues during motion. Hence, they can be employed effectively as an artificial nerve to in vivo repair neural defects. The appealing results of such stimuli-responsive hydrogel systems make them greatly promising for further nerve tissue regeneration applications.

#### 4.1.3 Dermal tissue engineering (wound healing)

Hydrogels act as scaffolds/matrices where cells are loaded, or encapsulated, and served with conditions appropriate for growth and multiplication as the presence of bioactive materials that encourage their adhesion and migration. That in turn shares in the effective reconstruction of the injured tissues [383,384]. Hydrogel polymeric scaffolds could provide the perfect choice for skin injury and epidermal regeneration. This unique ability was referred to their exceptional physico-biological characters as biocompatibility, biodegradability, biomimicry, as well as their in-situ crosslinking properties, adaptable mechanical, and swelling capacities [233,385].

Skin tissue engineering relied for years on many types of grafts including autogenic grafts and allogenic grafts. The increasing drawbacks related to these materials have drawn attention toward the search for harmless and biocompatible scaffolds like hydrogels. These restrictions were expressed by the giver site and include severe pain, frequent infections, and defective healing resulting in scarring over time [386]. The major limitation factors in the reconstruction of skin are the deficiency of autogenous skin and the non-compatibility problems encountered by allo- or xenografts. Thus, the implementation of tissue-engineered skin alternatives has been extensively extended in wound healing, particularly in burns [383,384].

Owing to their cytocompatibility, biodegradability, and their efficacy to enhance new cell growth and multiplication, smart/stimuli-responsive hydrogels have exhibited a substantial potential as wound dressings to enhance skin engineering applications. Among them, CH-based hydrogels that presented fascinating characters for wound healing referred to their superior bacteriostatic ability. Recently, Yang and Lin have designed a thermo-responsive CH-based hydrogel entrapping poly(propylene) (PP)-the g-AA-g-PNIPAAm nonwoven fabric used as wound dressings [348]. PP-g-AA-g-PNIPAAm CH has shown a satisfactory bacteriostatic property that could inhibit bacterial growth and support damaged skin healing. In another research, Rasool et al., have utilized CH to fabricate pH-responsive silver sulfadiazine-loaded CH/PVP as a system to deliver silver sulfadiazine at the location of skin injury to enhance its regeneration [349]. Carboxy methylcellulose (CMC) has been exploited by Soo-Hon Lee group to produce polymer–clay nanocomposite hydrogel films (PCNCHFs) composed of CMC, polyvinyl-pyrrolidone (PVP), and agar reinforced with nanosepiolite clay [350]. They were developed to achieve pH-sensitive release of laden 5-fluorouracil (FU) (PCNCHFs@FU) to the seat of skin injury. Lately, Zhang K. et al. [333], developed temperature-responsive GelMA based nanocomposite hydrogels for skin tissue engineering. This smart hydrogel nanocomposite system, GelMA-polydopamine (PDA)-aspirin (ASP) (GelMA-PDA-ASP) presented the remarkable thermo-responsive faster release of ASP at 40 ​°C higher than that at 25 ​°C and 37 ​°C. Moreover, it could attain successful healing in vivo and invitro (Table 1). These smart platforms have provided a unique, simple, and economic approach to fabricate novel systems that fit multiple biomedical applications including drug delivery, wound dressing, and tissue engineering.

#### 4.1.4 Corneal tissue engineering

Diseases of the cornea are serious sight-affecting conditions, that can result in cloudiness, alteration of corneal structure, scarring, and ultimately blindness. They are the second main reason for blindness [387]. Corneal transplantation is the key contemporary assigned solution for this issue. However, the supply of corneal transplants is limited [388]. Hence, the attention is directed towards designing alternative treatment modalities to meet this escalating request for corneal replacers.

Reinforcement of the innate corneal tissue using tissue-engineered polymeric constructs has received terrific attention. Ideal corneal tissue-engineered constructs must imitate the natural cornea and provide the optimum conditions for the protection of the living structures, while simultaneously provoking the formation of de novo tissues to replace the damaged ones [387]. Different decellularized biomaterials have been explored to preserve the structure and biochemical conformation of the cornea and were encouraging for the renewal of impaired corneal tissue. Among them, bovine and porcine decellularized corneal xenografts [388,389] that showed significant success as corneal transplants. However, their use is still challenging regarding immune rejection and disease transmission.

Hydrogel bioscaffolds provided a promising substitute for corneal bioengineering. For that intent, two categories of hydrogels were employed: collagenous (foams, gels, and sponges) and non-collagenous (gelatin, CH, keratin, and silk) matrices [387]. Transparency of hydrogels was an important criterion to support native corneal tissue regeneration via enhancing new keratinocytes and collagen formation and disseminating the moisture that consequently prevents swelling [390].

Recently reported temperature-responsive hydrogels are amongst the most frequently utilized hydrogels for multiple biomedical applications, particularly cornea regeneration [143,351,352]. Furthermore, Hamcerencu et al., have confirmed the capacity of Gellan maleate (MA-G) combined N-Isopropylacrylamide (NIPAm) (MA-G/NIPAm) hydrogels as unique, biocompatible ocular inserts for thermo-responsive release of ocular drugs (Adrenaline and Chloramphenicol) [143]. In an extra study, Chen and co-workers have presented CH-based hydrogel combined with disodium α-d-Glucose 1-phosphate (DGP) as a feasible thermo-sensitive hydrogel system for the release of levocetirizine dihydrochloride (LD) ocular drug [352]. The outcomes have confirmed the hydrogel's substantial ocular tolerance. Moreover, the fabricated hydrogel system (CH/DGP) has presented prompt initial release with efficient continual release of LD drug with marked improvement of LD cornea penetration. Lin et al., have integrated specific pathogen-free porcine platelet-rich plasma (P-PRP) to HA-based hydrogel combined with Pluronic F-127 (HA-F) [351]. This temperature-sensitive hydrogel system has been employed with high efficacy to share in the cure of corneal injuries via enhancement of the ocular endothelium regeneration.

Stimuli-responsive hydrogels with special regard to thermo-responsive hydrogels have provided satisfactory outcomes in eye tissues reconstruction. This ensures their high promises as potential players for the engineering of ocular tissues in the foreseen future.

#### 4.1.5 Osseous tissue engineering

3D hydrogels have exhibited favorable outcomes concerning support of bone defects healing and repair owing to their high angio-osteogenic potentials [177,391]. Although the capability of bone to heal spontaneously, the utilization of bioscaffolds as hydrogels plays a fundamental role to enhance the healing process, minimize the healing period, and thus, limit postoperative complications and maximize the success of treatment. Providing precise on-demand release of bioactive molecules at the targeted tissues is crucial to potentiate the objectives of regenerative medicine. That was attributed to the high harmony level of the biological cascades in tissues, especially bone [392].

In the context of bone bioengineering, the employment of US stimuli as on-demand triggers during fracture healing and callus disruption is exceptionally appealing because of their additional valuable osteoinductive influence towards enhanced bone regeneration [17]. In a recent study, Levingstone and co-workers have designed temperature-responsive P(Alg-g-NIPAAm) hydrogels blended with hydroxyapatite (HAp) [354]. These hydrogels have presented great US triggered capacity for on-demand release of multiple bioactive therapeutics like sodium fluorescein (NaF), bovine serum albumin (BSA), and bone morphogenetic protein 2 (BMP-2) essential for osteo-regeneration. In the same context, to enhance bone engineering, hemicellulose xylan has been combined with CH-based hydrogels to form hemicellulose xylan/CH composite hydrogels intended for the renovation of fractured bones [356]. Results revealed that these smart thermo-sensitive hydrogel composites were effective bone grafts for the repair of bone defects. In another elegant research by Zang et al., they could evaluate the capability of CH-based injectable hydrogel blended with β-glycerophosphate (CH/β-GP) for bone bioengineering [355]. The findings revealed the efficacy of CH/β-GP as cargo for bone morphogenetic protein-7 (BMP-7) and ornidazole (ORN) thermo-sensitive delivery for periodontal regeneration in class III bone defects in beagle dogs.

#### 4.1.6 Cartilaginous tissue engineering

Cartilages have a limited blood supply; therefore, they present a restricted self-healing potential, and the renewal of their defects is still challenging. The investigation has incredibly increased to find a promising substitute that can support cartilage injuries and defects healing and repair. Hydrogels were utilized as an effective minimal-invasive option for the remedy of big cartilage defects. They could provide optimum conditions vital for the restoration of the articular cartilages. Moreover, they could imitate the ECM owing to their mechanical characters, swelling behavior, and lubricating capacity [393]. In addition, hydrogels for 3D cell culture have been employed for cartilage bioengineering because of their high liquid-based structure. Hydrogels could be seeded by stem cells besides other proteins and growth factors necessary for cell growth and generation. Also, their porous framework could support cell transplantation and proliferation to the target site of the defect [391].

Smart/stimuli-responsive hydrogels have presented significant progress for the aim of chondro-engineering. Temperature-sensitive chitosan-g-poly(N-isopropylacrylamide) (CH-g-PNIPAAm) hydrogels were seeded with 3D Mesenchymal stem cells (MSCs) to obtain CH-g-PNIPAAm@MSCs hydrogels [357]. They could support in vitro proliferation and differentiation of cartilage from MSCs. Hence, they are promising scaffolds to fabricate biomimetic platforms intended for the engineering of cartilaginous tissues. In another in vivo study, Zhou et al., have fabricated transforming growth factor β1 (TGF-β1) laden poly(ε-caprolactone)–poly(ethylene glycol)–poly(ε-caprolactone) (PCEC) hydrogels. They provided a thermo-responsive release of TGF-β1 at the knee cartilage full-thickness defects in the rat model for proper chondrogenesis [358]. Liang et al., have introduced intelligent hydrogels with substantial dual sensitivity to pH and ionic strength. They were constructed from CH and carrageenan (CG) to form CH/CG composites used to enhance the chondrogenic differentiation of ATDC5 cells ex vivo [338]. Moreover, they provided outstanding promises for applications of chondro-engineering.

The implementation of smart/stimuli-responsive hydrogels in cartilage and bone tissue engineering has shown great advances. However, extra investigations are still required to study their capacity for clinical applications.

#### 4.1.7 Tendinous tissue engineering

Tendon tissues usually express a limited regeneration capacity because of the acellular nature of their structure. Consequently, existing repair modalities for tendon tears and injuries usually fate with leaving nonfunctional scar tissues that impair the mechanical abilities of the tendons with subsequent damages and injuries [394]. Tissue engineering practices have arisen as means to boost the engineering of tendon tissue. Diverse tissue engineering protocols including growth factors, scaffolds, cells, other bio-factors, and sometimes a mixture of them were adopted to improve the reconstruction of tendon tissues [394,395].

Scaffolds represent the most broadly studied approach in tissue engineering. Injectable hydrogels were recognized as potential promising scaffolds for tendon engineering. They potentially guide the tendon collagen matrix directly to the site of injury, supporting cell migration and healing. That role provides rapid healing and allows faster recuperation following prevalent orthopedic lesions with minimal possible complications [394]. These unique hydrogels can provide accurate, low, or non-invasive tendon regeneration modalities that can be carried to the injured part where they polymerize by body temperature, act as carriers of cells, proteins, or growth factors. Besides, they adjust the space/defect of the damaged tissue, provide a supportive collagen fibers nanostructure that provides structural stability, and prompt the healing procedure [396,397].

Stimuli-responsive hydrogels showed eminent prospects for the implementation in the engineering of tendinous tissues. Ping Chen's research group has successfully fabricated a temperature-responsive CH and HA crosslinked PNIPAM to obtain HA–CH–PNIPAM or HACPN hydrogel system [360]. This hydrogel could be utilized effectively to promote tendon regeneration with the prevention of post-operative peritendinous adhesion in vivo. Furthermore, Silva et al. could fabricate a magnetic-responsive hydrogel comprised of platelet lysate (PL) enriched methacrylated chondroitin sulfate (MA-CS) and entrapping iron-based superparamagnetic nanoparticles (MA-CS MNPs-PL) [359]. This smart, versatile, and multifunctional hydrogel has successfully guided the differentiation of both tendon-derived (hTDCs) and adipose tissue-derived (hASCs) mesenchymal stem cells towards the development of both tendon and bone-like matrices. This was attributed to their capacity to achieve magnetic-sensitive delivery of PL and release of PL-derived growth factors. However, with exceptional progress in the role of smart/stimuli-responsive hydrogels for tendon engineering, additional endeavors are still needed for their employment in clinical practice.

#### 4.1.8 Meniscal tissue engineering

The meniscus is a fibrocartilaginous structure that plays a pivotal role to preserve the functionality of the stifle joint. Like cartilages, menisci have a confined capability for spontaneous healing owing to their low or no blood supply. Consequently, it is extremely important to adopt the advances of tissue engineering to reconstruct the impaired meniscal tissues [340].

Due to their minimal invasiveness, the injectable hydrogels offered an acceptable option for repairing meniscus injuries. They could effectively deliver the cells, growth factors, proteins, or drugs laden on them to the area of meniscal tear and provoke their regeneration [398].

Thermo-responsive hydrogel, due to their availability, cost-effectiveness, facile cell and biomolecules loading to their surfaces, provision of minimally invasive surgical implantation with optimum defect filling ability, biocompatibility, and biodegradability, is one of the stimuli-responsive hydrogels for meniscal tissue engineering [399,400]. However, their mechanical and bioactive properties were subject to further investigations and improvement [401,402]. In that context, Chen et al., have cross-linked glycol chitosan (GC) and multialdehyde functionalized 4-arm (4-arm PEG-CHO) to develop hydrogel known as GC/4-Arm PEG-CHO hydrogel system [361]. This hydrogel system exhibited efficient temperature-sensitive release of TGF-β1 to improve the fibrochondrogenic differentiation of BMSCs ex vivo and promote the regeneration of meniscal defects in the rabbit model in vivo.

#### 4.1.9 Intervertebral disc disease regeneration

The main constituents of the intervertebral disc are the peripheral fibrous portion (annulus fibrosus) and the central jelly-like portion (nucleus pulposus). The core of the nucleus pulposus (NP) is comprised of a gelatin-like structure and water-rich material, together with loose collagen connective tissue fibers that resist the compression. This central structure's elasticity renders the vertebral disc able to endure elevated contortion and compression forces. It also expands its capacity to achieve other functions like rotation, flexion, cushioning, and maintaining a healthy spine function [403]. With age, the intervertebral disc components, due to loss of their water content, loss their elasticity and become stiffer. Therefore, the disc becomes more prone to degeneration due to the loss of its ability to adapt the compression. Consequently, other unfavorable sequels as herniation of the NP can occur and result in compression of the surrounding nerves [404].

Intervertebral disc degeneration (IVDD) or degenerative disc disease (DDD) is a middle-aged predominant affection that is usually associated with persistent back ache. The first stage of degeneration usually entails the NP. Thus, prompt build-up and revival of this part will assist to avoid further disintegration of the annulus fibrosus. The existing treatment strategies of IVDD are not just proved to be ineffective in the long run, however, they additionally may result in the involvement of the neighboring intervertebral discs. To curb the advancement of this disease, alternative regeneration approaches are necessary to counteract the degeneration and enhance the renewal of impaired tissues [405]. The ideal core substitute ought to be inert, hydrophilic, viscoelastic, and space-filling to suit the feature of the natural nucleus. On the other side, for this material to match the surgical approaches, it should likewise be minimally invasive, injectable, and radiopaque [406].

Hydrogels, with emphasis on injectable and bio-adhesive hydrogels, have been presented as an effective substitute to the NP for the repair of IVDD and have displayed a substantial potency for NP regeneration [404]. They are exceptionally hydrated polymeric structures, conformable, strong, viscoelastic, porous, and generally comprised of water. Processed natural hydrogels have exhibited significant priority to the synthetic ones for being less cytotoxic, cost-effective, fit for diverse applications of tissue engineering, and mediate cell adherence and migration [406].

**Fig. 10:**
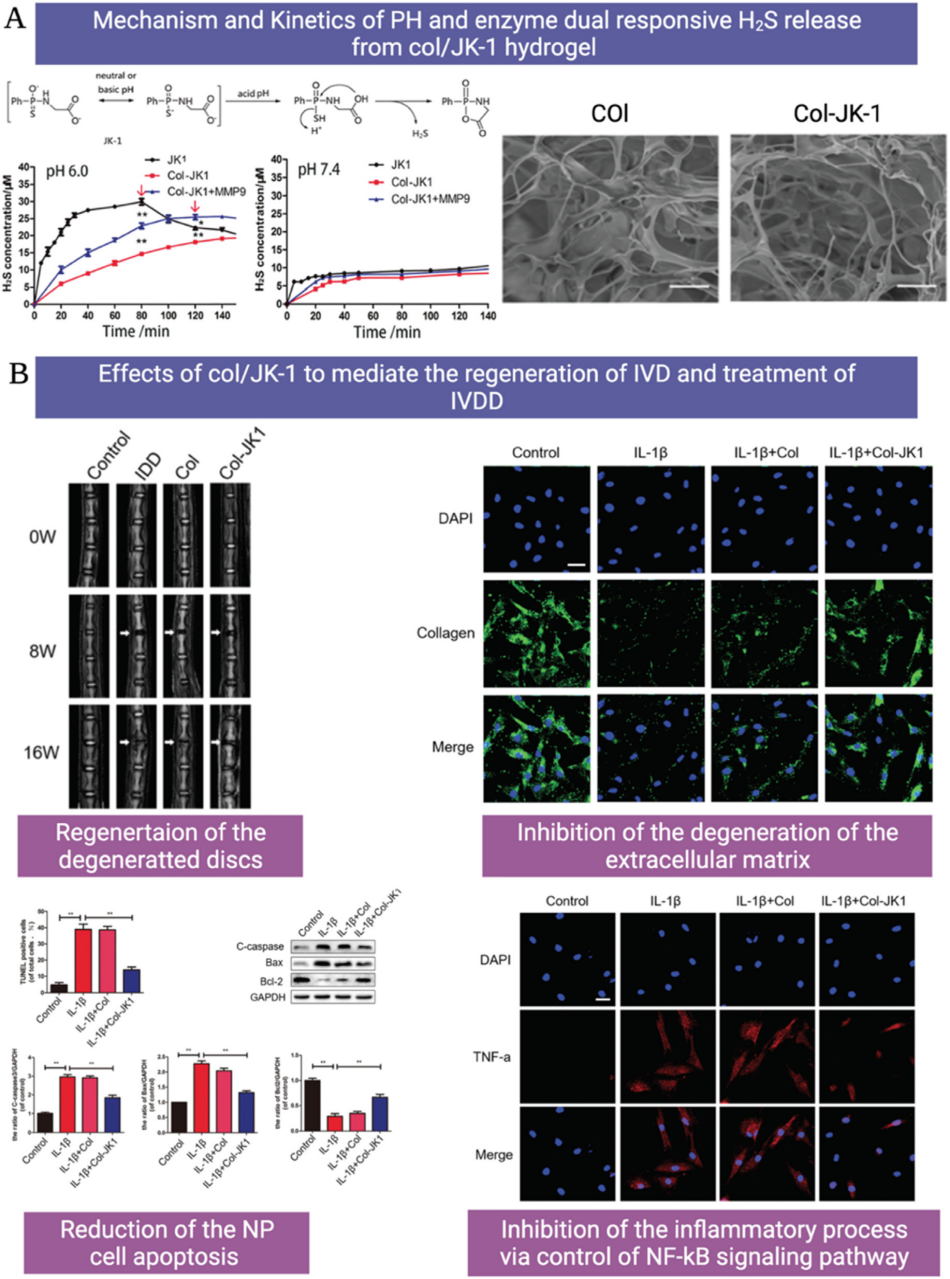
Schematic illustration of pH and enzyme dual responsive hydrogel (Col-JK-1) for release of H_2_S to treat IVDD. (A) Mechanism and kinetics of pH and enzyme responsive H_2_S release from Col-JK-1 hydrogel (MMPs were represented using MMP9 (50 ​mg ​mL^−1^)). (B) Effects of col/JK-1 to mediate the regeneration of IVD and treatment of IVDD showed marked regeneration of the degenerated disc. Besides, inhibition of the ECM degeneration, the apoptosis f Nucleus Pulposus (NP), and the inflammatory reaction. Copyright © 2019, The Royal Society of Chemistry. Replicated with permission from Ref. [337].

Smart/stimuli-responsive hydrogels have shown outstanding potential for treating IVDD. Feng and co-workers have encapsulated miRNA/PGPC Polyplex in PEG hydrogels (miRNA/PGPC@PEG HG) to achieve two-stage enzyme (MMPs) sensitive miR-29a delivery to the NP cells and promote engineering of degenerated IVD [362]. This novel approach is promising for IVD tissue engineering and the treatment of chronic IVDD. Zheng et al., have designed a dual pH and enzyme-responsive hydrogel system to deliver hydrogen sulfide (H_2_S), a promising drug candidate for control of many degenerative diseases via sharing in many physiological and pathological mechanisms, to the area of disc degeneration [337]. Upon injection, this hydrogel scaffold named Col-JK1 has presented effective acidic pH and enzymes (MMPs)-responsive release of H_2_S in the pathological IVDD environment. The Col-JK1 hydrogel could effectively share in the degenerated disc regeneration, impede the disc degradation, and inhibit the apoptosis of NP attributed to its capability to suppress the inflammation via regulation of the nuclear factor-kappa B (NF-kB) signaling pathway as demonstrated in Fig. 10.

### 4.2 Smart/stimuli-responsive hydrogels for tailored/controlled drug delivery

Regarding drug delivery, the loading/release capacity of conventional hydrogels is still poor for some drug compounds, especially hydrophobic ones. Subsequently, different trials including polyampholyte, twofold system, and nanocomposites have been conducted to improve their mechanical power, and drug discharge abilities, besides upgrading their electrical conductivity behaviors [407]. Injectable hydrogels are desired for many biomedical applications, especially drug delivery and tissue engineering owing to their promising 3D carrier property, biocompatibility, minimally invasive, and compliant form for administration [408].

Hydrogel drug release devices can provide a favorable therapeutic outcome in the clinical field. Traditional drug administration methods often require large amounts or frequent administrations to be therapeutically effective. Thus, the demerits of harmful effects and poisonousness become more frequent [34]. Since their physical, mechanical, and biodegradation assets can be adjusted, hydrogels can afford an appropriate platform for several physicochemical reactions with the loaded drugs. subsequently, they can support strict control of therapeutics release rate to the targeted delivery site [193]. Moreover, Hydrogel drug delivery platforms can regulate the way of the accessibility of drugs to the cells. Consequently, they were employed in diverse medical branches including cardiology, immunology, oncology, and pain control. Hydrogel aqueous structure plays a decisive role to minimize the possibility of drug degradation and accumulation when exposed to solvents. Hence, they are able to get high loading of water-soluble drugs [44]. Furthermore, they can encapsulate water-insoluble drugs [409].

The cross-linked polymeric configuration of hydrogels avoids the penetration of different proteins. Moreover, it is thought to prevent premature disintegration of the bio-therapeutics. Hydrogel-based drug release devices are attracting more attention in current years and their impact is going to upsurge in the future as a promising in situ drug delivery platform [96,181,408].

**Fig. 11:**
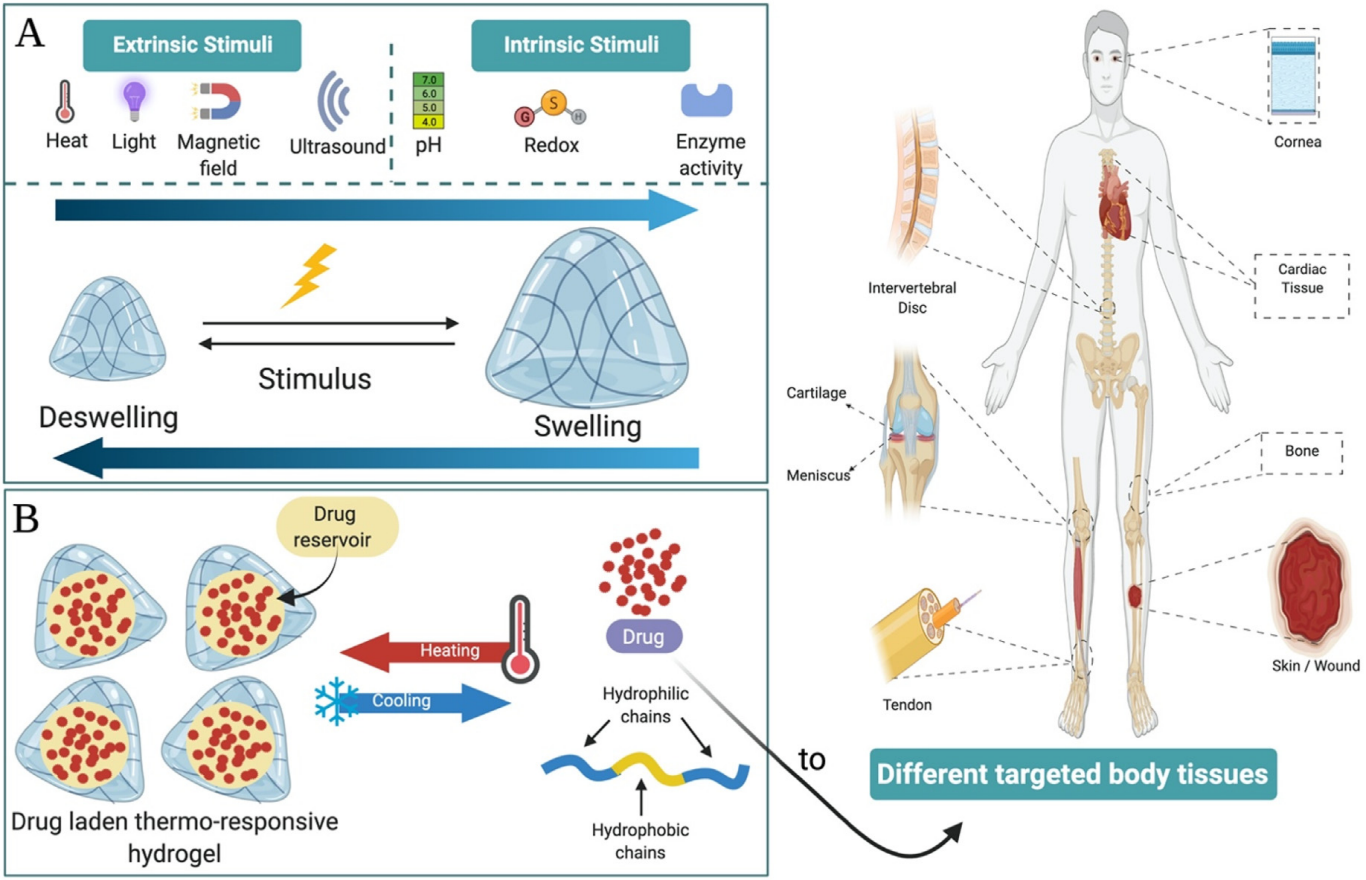
Mechanism of stimuli-responsive drug release from smart hydrogel scaffolds. (A) Different stimuli provoke Swelling-deswelling behavior. (B) Mechanism of thermo-responsive drug release to different targeted body tissues from a temperature-responsive drug-laden hydrogel.

Upon exposure to different extrinsic and intrinsic stimuli, a marked deformity is triggered in the stimuli-responsive hydrogels in the form of swelling that facile the release of drugs and/or other biomolecules encapsulated within their networks as illustrated in Fig. 11. This functionality provides precisely targeted delivery of these components at desired time and site [410]. Smart/stimuli-responsive hydrogels have been utilized as efficient delivery systems for many bioactive molecules and therapeutics in multiple applications in biomedicine. They included, but were not limited to, hybrid electro-responsive hydrogels for enhancement of wound healing [411], dual temperature and pH-responsive hydrogels for treatment of cancer [[412], [413], [414], [415]], and magnetic-responsive hydrogels for control of neural (Parkinson) disease [416]. Recently, the field of nanotechnology has emerged significant progress with the ability to produce nanoparticles from different sources with substantial promises in diverse biomedical applications including drug delivery [417]. The combination of nanomaterials with stimuli-responsive hydrogels has endowed targeted drug delivery, minimized possible risk factors, and improved therapeutic outcomes [418,419]. Smart/stimuli-responsive hydrogels have confirmed remarkable findings regarding drug delivery applications. However, many recent studies were conducted on ex vivo models. Their exploitation in the in vivo models for clinical practice is still under investigation with great endeavors still requested in this track.

### 4.3 Smart/stimuli-responsive hydrogels as smart biosensors

Biosensors are highly sensitive analytical devices with numerous advantages including high sensitivity, specificity, cost-effective manufacturing, simplicity, portability, and short response time [420]. They are produced via merging materials responsive to the adjacent stimuli as temperature [421,422], light [423], and ionic strength and pH [424,425]. Implementation of smart/stimuli-responsive hydrogels in biosensors includes three main categories: i) signal recognition, (ii) signal transfer to the gauging electrode, and iii) tailoring the response to the signal [426,427].

Recently, biomimetic hydrogels have been extensively exploited to build biochemical sensors thanks to their unique responsive ability to the neighboring stimuli. Thus, meaningful advancement in the control of several diseases has been attained as they can experience phase alteration mechanics and transfer the biological information to these sensors [151,428].

Specially designed, enzyme-free hydrogels were prepared to function as chemo-mechanical transducing probes in piezo-resistive biosensors through their response to metabolic alterations. The fundament depends mainly on the monitor and assessment of the hydrogels’ response to solution different glucose concentrations that consequently enable the sensors to identify the metabolic alterations [331]. These biosensors are wished to share effectively in achieving perpetual measurement of blood sugar in diabetics.

Studies have used CH and cellulose for biosensors applications [429,430]. In that context, Qiu et al. have reported that cellulose-based smart materials, owing to their exceptional stimuli-responsive behavior, have vast applications in the sensing field [430]. Leu et al., have designed a microscopic, pillar-like smart hydrogel network with improved surface area/volume ratios constructed in microfluidic networks. Once the pillars face their target, molecules existing in the liquids, exhibit swelling-deswelling responsive reactions. Consequently, change in resistance can be investigated using a potentiostat [339]. These smart hydrogels have afforded inexpensive, yet prompt responsive models for biosensing applications. However, the conflicting time of swelling response is usually encountered as a conceivable challenge.

### 4.4 Smart/stimuli-responsive hydrogels as actuators

Stimuli-responsive hydrogels, with their unique power to exhibit significant, yet reversible fluctuations in their volume in response to the intrinsic or extrinsic stimuli, have been employed to develop actuator models that can imitate different aspects of the alive systems [431]. This capacity to present reversible ‘on-off’ responsive swelling behavior to the physiologically pertinent signals has paved the way to utilize stimuli-responsive hydrogels to design sensors, micromanipulators, robotics, and optical systems with adjustable focal length to manage the liquid flow in microfluidic systems [332,432].

Even though the usage of smart/stimuli-responsive hydrogels as actuators had attracted great attention owing to their substantial swelling-deswelling stimuli-responsive characters, their aptitude to be actuated improves the functionalities which can be created by every actuator, and expedite the design of microfluidic devices. This technology is yet at the beginning phase of improvement. Furthermore, these intelligent hydrogels present a promising potency to be employed in different biotechnological progressions and microengineering items [331]. Several stimuli-responsive hydrogels have been employed for the development of robotics and actuators that can present programmable actuation in response to multiple stimuli (pressure, electro-magnetic stimuli, temperature, light, and pH). The engineered smart hydrogels can attain simple bending and twisting, as well as 3D micro and macro shape transitions with diverse functionalities for many biomedical applications [[433], [434], [435], [436], [437], [438], [439], [440], [441]]. For example, to improve the retention capacity, targeting precision, cell survival, and the efficacy of tissue regeneration, Yasa et al., recently developed magnetic responsive 3D printed microactuators. The devices could recapitulate the physical and biochemical characters of the stem cell niche to achieve unique targeted cell delivery. Moreover, they could guide the cell differentiation towards the preferred lineages with fine, and remotely controlled cell localization [178]. In another work, magnetic-responsive hydrogel nanocomposites were employed to fabricate actuators in the form of biodegradable microswimmers. They could effectively present tailored enzyme-responsive drug release. Moreover, these systems could also provide a diagnostic function via sensing certain pathological markers for cancers. Hence, they accelerate the drug release to the tumor site with a unique harmonized theranostic property [179]. Magnetic nanoparticles were combined with PEDGA hydrogels to design outstanding actuators. The fabricated systems provided programmed control of the microactuators. Besides, they presented promising potentials for future implementations at the cell level as organ-on-a-chip and other biomedical applications [173]. The significant progress in the designing strategies of stimuli-responsive hydrogels for the fabrication of smart actuators and robotics is a pivotal step to pave the way for their rebound toward diverse biomedical applications.

## 5 Smart/stimuli-responsive hydrogels for 3D and 4D bioprinting

Smart/stimuli-responsive hydrogels provide supportive and regulatory platforms for the cells entrapped within their networks during 3D bioprinting. Smart hydrogels and 3D technology present a stout integration for the printing of multidimensional (3D and 4D) structures [442]. Currently, reactions of hydrogels, as deformation and contraction, caused by cellular activities as migration, proliferation, traction, and dispersal have been measured by computational models [3]. The distinctive stimuli-responsive aptitude of intelligent hydrogels affects the cells incorporated in their matrices.

The characters of hydrogels that are fundamental in deciding the modeling of tissue renewal and cell fate at a semi-constant condition, change over time. Hence, time is another factor critical to perceiving the cell-material interaction dynamics. This indicates the necessity to acknowledge progressive changes in viscoelastic characters of the substrate during defining the shape of the tissue [442,443]. PNIPAAM hydrogels are one of the stimuli-responsive platforms that can be utilized for 3D printing applications and show volume alterations (swelling/deswelling) responsive to specific stimuli. Hence, they can be employed for many biomedical purposes including drug delivery and tissue engineering [444,445]. For example, Polycaprolactone/alginate 3D printed stem cell-laden tubular scaffolds have been fabricated to repair the spinal cord tissue [446]. A hybrid Gelatin Methyacrylate (GelMA)/PEG hydrogel scaffold was utilized to develop an anisotropic stem cell niche for designed differentiation of embryoid bodies (EBs) for neural tissue engineering [447]. To improve the stability of poly(ε-caprolactone) platform for the reconstruction of the intervertebral disc, Gloria, et al. [405], utilized collagen/HA hydrogels. Moreover, Shafiee et al. [448], fabricated poly(lactic-co-glycolic acid) microspheres loaded hydrogel platform incorporating differentiation factors to reproduce a niche for the growth of stem cells. Furthermore, many smart/stimuli-responsive hydrogels have been exploited in 3D bioprinting for further diverse biomedical applications [173,175,178,179].

Despite the great advances in 3D bioprinting techniques, many challenges are still present. Among them are the printing strategies, multifactorial process to choose the appropriate bioink material, design of the scaffolds, cell vitality, mechanical and heterogenic characters of the printed scaffolds [449]. Moreover, the 4D bioprinting approach presents a time factor that enables the 3D printed scaffolds to present stimuli-responsive modulation of their shape and/or function. Hence, these scaffolds are more appropriate for applications in the biomedical and tissue regeneration fields with substantial capacity to substitute the native structures [450,451]. Many smart/stimuli-responsive hydrogels, attributed to their outstanding responsive capacity to extrinsic stimulations, have been widely used in 4D bioprinting applications. For instance, PNIPAAm was employed to fabricate 4D printed thermo-responsive smart valves. The device (Alg./PNIPAAM) was mechanical robust and thermally actuating [452]. In another work, stereolithography technique was used to produce 4D printed dual temperature and hydration responsive grippers. The device is composed of poly(N,N-dimethyl acrylamide-co-stearyl acrylate) (P(DMAAm-co-SA))-based hydrogels with variable concentrations of SA crystalline monomer within the SMG network. This system is potential for different applications as encapsulation devices, biomimetic actuators, and/or soft robotics [453]. Other stimuli-responsive hydrogels including ionic strength [454,455], electric [456], light [457], and magnetic-responsive [458] hydrogels have been exploited to fabricate 4D printed devices that present substantial capacities for diverse biomedical applications.

Alterations in the features of smart/stimuli-responsive hydrogels will influence the evolution of the engineered tissues. This area is subject to further future investigations.

## 6 Smart/stimuli-responsive hydrogels for 3D cell culture

Hydrogel platforms, owing to their hydrous polymeric structure and resemblance to the normal tissues, can be developed to regulate the cell destiny and occupation that is a major notion in the domain of tissue engineering. They were utilized as carriers of cells for uniform cell loading in 3D cell culture [15,459].

Inside the tissues, cells are surrounded by a complicated 3D ECM microenvironment. Hence, it is indispensable for the ideal biomaterials intended for 3D cell culture to provide conditions mimicking that of the connate tissue [15]. Synthetic hydrogel scaffoldings are distinguished by being biodegradable, biocompatible, and having the power to function as effective matrices for cutting-edge tissue engineering implementations. These matrices also provide a master network for cell adherence, migration, and multiplication. Likewise, they provide structural stability vital for cell self-assembly [459]. Such systems fit a broad spectrum of applications in the biomedical scope, mainly in elevated-throughput drug inspection [460,461], cell biology [15], cancer biology [[462], [463], [464], [465], [466], [467], [468]], disease modeling [469,470], and tissue engineering [459]. Collectively, hydrogel matrices not only present encouraging results for 3D cell culture but also exhibit high promising potentials for future broad range applications in the biomedical fields, chiefly tissue engineering [471].

The evolution of smart/stimuli-responsive has enabled better imitation of the dynamics of ECM. They possess the aptitude to modify their physicochemical characters responsive to different external and internal stimuli [472]. This makes them fit various biomedical applications. Fisher S. A. et al., fabricated 3D HA hydrogels that were responsive to MMPs enzymes secreted by breast cancer cells. These intelligent platforms presented the remarkable ability for invasion of the breast tumor cells superior to that of other conventional hydrogels or other MMPs-responsive scaffolds as PEG [473]. That was referred to the high biomimetic capacity of HA to recapitulate the microenvironment of the tumor cells as it was highly expressed in the tumor ECM. Moreover, the cancer cells usually upregulated the HA receptor (CD44) [[474], [475], [476]]. Furthermore, MMP-responsive hydrogels have been exploited in diverse biomedical purposes as cardiac tissue engineering [477], osseous tissue engineering [478], and stem cell biology [479]. However significant breakthroughs in the utility of smart/stimuli-responsive hydrogels for 3D cell culture purposes. The combination of spatially specific chemical signals, a temporally regulated trigger of bioactive molecules, and spatiotemporal modulation of hydrogel rigidity with stimuli-responsive hydrogels will produce a single scaffold that will be a more sophisticated biomimetic device with a higher ability to effectively share in further deep in vitro investigation of significant biological issues.

## 7 Smart/stimuli-responsive self-healing hydrogels for biomedical applications

Hydrogels intended for self-healing are produced via different covalent and non-covalent bonds in addition to some interactions as hydrogen bonds, hydrophobic and electrostatic interactions. The crosslinked hydrogel polymers have been dedicated to healing functions in the biomedical field [150,233]. They are among the leading biomaterials designated for prospects of wound healing. They can share effectively in the restoration of the structure and occupation of the damaged tissues. These biomaterials can compensate for their old broken bonds by newly formed ones, besides adapting to the surrounding changes [343]. Depending on their tensio-metric characters in biomedical fields, self-healing hydrogels may be either soft or robust [480]. Soft ones are injectable and consequently employed in targeted drug/cell delivery and 3D printing purposes. Unlike, the robust members are represented as soft robotics including either implantable biosensors with prolonged lifetime or biosensors with mechanical properties for repairing the damage or relieving the fatigue [480].

Self-healing hydrogels cover a massive scope of biomedical applications as tissue engineering [481,482], drug delivery [343,483], wound healing [442,484], and 3D bioprinting [442,443].

Great endeavors have been conducted to enhance the capacities of self-healing hydrogels via the production of stimuli-responsive self-healing hydrogels. Consequently, widespread their implementation in the biomedical field. Guo B. group has designed a multifunctional β-cyclodextrin (β-CD) based poly(NIPAM-co-β-CD)/CNT/PPY hybrid hydrogel system that has displayed substantial dual temperature and NIR light-responsive capacities. Moreover, they presented an outstanding potential for diverse biomedical applications including intelligent pressure and motion sensors, and self-healable smart electronic devices [485].

On the other side, many stimuli-responsive self-healing hydrogels were utilized for therapeutic purposes as drug release vehicles. Bilalis et al., have fabricated another smart hydrogel composed of pentablock terpolypeptide of the type PLys-b-(PHIS-co-PBLG)-PLys-b-(PHIS-co-PBLG)-bPLys and encapsulated with gemcitabine [341]. The hydrogel worked as a PH and enzyme-responsive cargo for the conveyance of gemcitabine drugs to treat pancreatic cancer. Another research group has recently employed methylcellulose/chitosan (MC/CH) dual-crosslinked DAMC and a water-soluble chitosan oligomer (DAMC/CHI–O) copolymer hydrogels for temperature-sensitive delivery of biomolecules as vitamin C (l-ascorbic acid) and adenosine for cosmetic purposes [486]. The attractive results of smart/stimuli-responsive self-healable hydrogels have made them highly encouraging to be implemented in various applications in tissue engineering, cosmetics, and drug delivery.

## 8 Conclusion and future perceptions

Over the ultimate few decades, massive investigations have been conducted to develop more refined hydrogel-dependent scaffoldings for implementations in biomedical and tissue engineering fields. Consequently, a paradigm shift in the planning, fabrication methods, characters, and applications of smart/stimuli-responsive hydrogels has been displayed. The focus of ongoing research is to upgrade the biological and mechanical features of hydrogels via modifying their polymeric design. This renders them one of the leading matrices utilized for the intentions of healthcare. Frequent endeavors were accomplished to fabricate smart/stimuli-responsive hydrogels for explicit applications through careful study of their encompassing microenvironments and exploiting these efficient enhancements for further utilization.

In the current review, recently fabricated hydrogels and their designing approaches have been outlined. Different approaches have been employed to synthesize smart/stimuli-responsive and dynamic hydrogels. However, many intricacies have been declared correlated to the mimicry of hydrogels to the native 3D structures. In the future, the development of multi-stimuli-responsive hydrogel platforms imitating the native 3D structure is a potential area for investigation. The development methods of 3D-designed hydrogels inevitably demolished their biocompatibility and hydrophilicity. These research expansions encompass the combination of diverse polymers for the development of different hydrogels, which similarly points to the elaboration of novel technicalities of the extrinsic stimuli-responsive hydrogels. Additionally, the fabrication of smart/stimuli-responsive hydrogels with coveted robust and efficient fundamental physicochemical characters is yet under investigation. Likewise, the fabrication of novel smart hydrogels with programmed, even complicated self-bending, twisting, and folding attitudes is another remarkable subject for future research.

Although the significant progress in the hydrogel bioengineering and fabrication approaches, biodegradability, reactivity, microstructures, surface hybridization, inflammatory and immune reactions are still encountered as potential challenges during their manufacture. Hence, more attention should be directed toward the synthesis of hydrogels that can control and minimize the immune response. The utilization of different solvents during the synthesis of smart platforms presents an additional threat of toxicity. Thus, the amalgamation of natural polymers during hydrogel crosslinking is an important subject for further research in the future. The future in-depth study of the hydrogel biodegradation and the adjustment of its level with cell multiplication and adherence is also crucial. Future research should focus also on the scaling of the size of the hydrogel to the level of single-cell to enable individualization of the sequencing data on a cell-by-cell level. This is crucial for the requisition of information regarding the biological subsets in the sample. Besides, the tailor of the release rate and speed of drugs and biomolecules from the smart hydrogels is very crucial for smart adjusted drug delivery. So that, future investigations should emphasize the production of sophisticated electro-responsive hydrogels with a more prompt responsive release.

Antigen or antibody-responsive hydrogels are outstanding smart materials for many uses in biomedical diagnostic. They exhibit unique swelling-deswelling transitions responsive either to antibody or antigen. Yet, the fabrication of smart hydrogels responsive to antigen-antibody interaction has not been reported. Therefore, this point is attractive for future research. Besides, many investigations declared the functionality of antibody-conjugated hydrogels. However, the fabrication of smart responsive hydrogels with antibodies in their backbone structure is still a question for future research.

In the foreseen future, based on the successive enhancements of stimuli-responsive hydrogels’ structure and consequently their function, the cell-scaffold interactions will be more obvious and pave the track to employ such attractive platforms for new tissue regeneration intentions. These intelligent hydrogels can afford a safer, successful, and potential alternative for many implementations in biomedicine including but not limited to early detection of diseases, disease control, and tissue engineering. Furthermore, many investigations are still required for their further implementation in clinical applications.

## Author contributions

El-Husseiny H. M. and Mady E. A. equally contributed to this work. El-Husseiny H. M. conceived the idea and discussed it with other authors. El-Husseiny H. M. and Mady E. A. wrote the original draft and prepared the figures. El-Husseiny H. M., Mady E. A., Hamabe L., Abugomaa A., Shimada K., Yoshida T., Tanaka T., Yokoi, A. revised the manuscript. El-Husseiny H. M., Elbadawy M., and Tanaka R. supervised the work, revised the manuscript, and edited the draft. All authors have read the manuscript and agreed to the submission.

## Funding

This article did not receive any kind of financial support.

## Declaration of competing interest

The authors declare that they have no known competing financial interests or personal relationships that could have appeared to influence the work reported in this paper.
